# Recent Advances in Wearable Healthcare Devices: From Material to Application

**DOI:** 10.3390/bioengineering11040358

**Published:** 2024-04-06

**Authors:** Xiao Luo, Handong Tan, Weijia Wen

**Affiliations:** 1 Department of Physics, The Hong Kong University of Science and Technology, Hong Kong 999077, China; xluoay@connect.ust.hk; 2 HKUST Shenzhen-Hong Kong Collaborative Innovation Research Institute (SHCIRI), Futian, Shenzhen 518060, China; 3 Department of Individualized Interdisciplinary Program (Advanced Materials), The Hong Kong University of Science and Technology, Hong Kong 999077, China; htanaf@connect.ust.hk

**Keywords:** flexible wearable devices, flexible substrates, healthcare monitor, self-healing materials, vital signal

## Abstract

In recent years, the proliferation of wearable healthcare devices has marked a revolutionary shift in the personal health monitoring and management paradigm. These devices, ranging from fitness trackers to advanced biosensors, have not only made healthcare more accessible, but have also transformed the way individuals engage with their health data. By continuously monitoring health signs, from physical-based to biochemical-based such as heart rate and blood glucose levels, wearable technology offers insights into human health, enabling a proactive rather than a reactive approach to healthcare. This shift towards personalized health monitoring empowers individuals with the knowledge and tools to make informed decisions about their lifestyle and medical care, potentially leading to the earlier detection of health issues and more tailored treatment plans. This review presents the fabrication methods of flexible wearable healthcare devices and their applications in medical care. The potential challenges and future prospectives are also discussed.

## 1. Introduction

Due to the increasing concern about health and the scarcity of medical labor, the need for real-time and rapid diagnostic tests is becoming increasingly apparent [1,2,3]. Wearable sensors have shown strong application potential in the medical field, such as real-time monitoring, in vitro diagnosis, body composition analysis, and energy harvesting, obtaining detailed information about the body and helping doctors to diagnose patients and carry out accurate and timely treatment [4,5]. Wearable sensors provide a novel and convenient means of diagnosis and treatment and, to a certain extent, reduce the high cost of hospitalization and the number of visits to the hospital and also, for some patients who are afraid of hospitals, provide a reassuring measurement means to obtain an accurate heart rate, blood pressure, and other data.

Basically, wearable sensors can be divided into two categories: (a) physical sensors and (b) chemical sensors [6]. Biosensors are an emerging interdisciplinary field in which a wide range of technologies can be found. However, it has also been reported that wearable electrochemical sensors have attracted less attention than physical sensors over the past decade [7]. Most biosensors directly measure changes in physical quantities, such as strain, pressure, displacement, velocity, and acceleration, etc.

Thanks to the rapid development of engineering technology, electronic technology has expanded wearable healthcare device measurement and application range. Flexible wearable devices have three main parts: a substrate, an active element, and an electrode [8]. From early rigid biosensors to later flexible sensors that can be attached to jagged skin surfaces, wearability is no longer limited to specific areas of the human body. Most of these devices can be worn in the teeth, mouth, neck, chest, arms, wrists, ankles, legs, and other areas, and can continuously provide real-time monitoring of various body information, including temperature, heart rate, blood oxygen, blood pressure, calorie consumption, step count, gestures, swallowing and so on. In addition, some wearable sensors can be directly fixed onto clothing [5]. Due to the diversity of human skin, sweaty environments, stretching, and strenuous exercise, wearable sensors must overcome these challenges. It is crucial to choose a suitable material, which requires softness, stretchability, comfort, portability, breathability, good biocompatibility, and cost economy [9]. These materials offer wearable healthcare devices a broader range of applications. 

At present, many reviews have reported on wearable sensors, but the details of the materials and applications still need to be integrated. In addition, the rapid development of materials in recent years has also promoted research outcomes of multiple types of biosensor devices based on multiple detection principles in the healthcare field and it is necessary to organize and review them. In this review, we analyze the types of materials, such as polymers, liquid metals, composites, etc., used in wearable medical sensor devices and report current research hotspots on biodegradable materials and self-healing materials in detail. We introduce their definition, performance, advantages, potential applications, and challenges. In addition, we report on the latest applications of wearable devices over the past few years, introducing the principles of wearable devices and their applications in healthcare. In the final section, we present the challenges and potential problems of wearable devices. We expect this review to generate widespread interest and promote the development of wearable sensors in multi-disciplinary fields.

## 2. Materials and Methods

Manufacturing flexible wearable sensors is limited by material and film thickness. Functional printing technologies are commonly used to process flexible sensors, including inkjet printing, roll-to-roll gravure printing, screen printing, 3D printing, stamp printing, and lithography. In recent years, there has been extensive coverage of these technologies [10,11,12,13,14,15,16,17]. 

Wearable biosensors are in direct or indirect contact with the skin during use, which requires that the material does not pose an additional threat to health and ensures comfort. Most wearable sensors are in direct contact with the skin for a long time, and biocompatibility also needs to be considered [18]. Biocompatibility is a property of living tissue that reacts to an inactive material, generally referring to the compatibility between the material and the host, according to the International Standards Organization (ISO). Substrate is the key to sensor comfort and materials such as metal foil, polymer, silicone, rubber, and so on are commonly used [6,19,20,21,22]. In general, the materials of biosensors can be divided into: (1) inorganic materials based on metal, carbon, and oxide materials; (2) organic materials such as polymers, small molecules, and natural biomaterials; (3) compound/mixed material in order to realize the function and structure of complementary properties [23]. However, inorganic materials tend to lack mechanical flexibility, and organic materials also face the challenges of poor stability and potential health threats [24]. In addition, wearable biosensors based on active nanomaterials need to focus on biocompatibility to determine the host’s immune response to the material and dose issues under different contact methods such as skin contact, wound contact, and microneedle invasion, etc.

This section will introduce the representative substrate materials for wearable, flexible devices, including polymer categories such as polydimethylsiloxane (PDMS), polyimide (PI), and polyurethane (PU), as well as environmentally friendly degradable materials and self-healing materials, which have been the focus of research in recent years.

With the rapid development of polymers in recent years, a variety of biosensors based on synthetic polymers have been manufactured, thanks to their excellent flexibility, energy supply, and electrical conductivity. Polymers used in biosensors can be divided into thermoplastic polymers, thermosetting polymers, elastomers, liquid crystalline polymers, polymer gels, piezoelectric polymers, intrinsically conductive polymers, and polymer composites, etc.

### 2.1. Elastomers

Elastomers exhibit rubber-like elasticity behavior. One of the most commonly used elastomers is PDMS, which is widely used in microfluidic chips, micropumps, electronic skin, and wearable sensors [25]. PDMS and its composites are common flexible substrates [26] with good chemical inertness, stability over a wide temperature range, variable mechanical properties, transparency, and the ability to define bonded, unbonded areas with ultraviolet light [27], which is essential for bonding electronic materials to the substrate surface.

**Figure 1:**
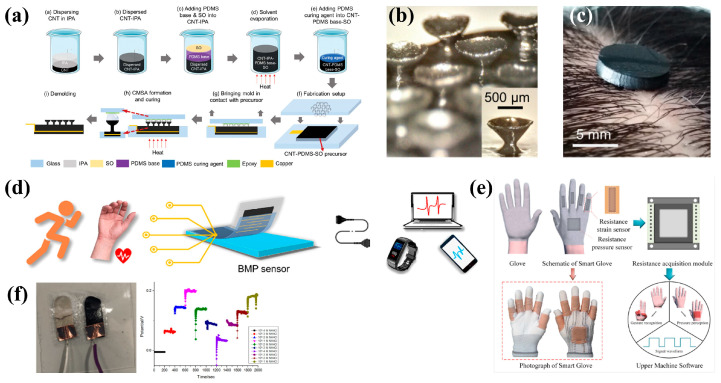
Flexible wearable healthcare devices on PDMS. (**a**) flow chart shows the fabrication of the CMSA sensor; (**b**) the microstructure of CMSA sensor; (**c**) the CMSA sensor can stick to the scalp to measure the EEG. Images reprinted from [28] with permission; (**d**) schematic diagram of the thin PDMS film-based sensor applied for portable human physiological signal acquisition. Images reprinted from [29] with permission; (**e**) image shows the integration of the resistive sensors with a smart glove for gesture recognition and perception detection. Images reprinted from [30] with permission; (**f**) adhesion between the electrodes and the PDMS composite substrate after the tensile test proved better than using pure PDMS. Images reprinted from [31] with permission.

Flexible sensors based on PDMS substrates can be manufactured using conductive materials, such as silver nanowires (AgNWs), silver nanoparticles, graphene, graphene oxide (rGO), carbon nanotubes (CNTs), and carbon black (CB), etc. Zhang et al. [28] reported a type of adhesive wearable sensor on hairy scalps. Measuring electroencephalogram (EEG) requires the instrument be in close contact with the scalp. However, compared to the arm, face, and other skin, the scalp is covered with a lot of hair, significantly affecting the sensor’s bonding effect. They designed and developed a composite sensor based on CNT-PDMS with a surface consisting of a series of conical microstructure arrays (CMSAs), which are suitable for the complex scalp hair environment. The CMSA sensor is made by the viscosity-controlled dip-up process (VCDP), which involves dipping a third of the radius of an array of glass beads fixed to a glass slide into a small pool of multiwall carbon nanotube-PDMS-silicone oil tuned to the correct viscosity (Figure 1a). The conical microstructure is formed by gently lifting and pulling the touch. After curing the precursor at 100 °C, the mold is removed to obtain CMSAs. Because of its small size, only a small amount of conductive gel is applied to each cone head, and the air between the scalp and the sensor can be gently squeezed out to attach to the scalp (Figure 1b,c) and accurately measure the EEG. It does not cause any skin irritation up to 6 h of wear, which shows that it has good biocompatibility. In addition, biomimetic PDMS substrate with surface patterning is also a research hotspot. Wang et al. [29] reported a kind of PDMS thin film substrate with a surface biomimetic microstructure constructed and Ti_3_C_2_T_x_MXene/bacterial cellulose (BS) as an induction layer with a flexible wearable sensor (Figure 1d). The complementary design of PDMS and BC/MXene intercalation can adjust the compression rate and improve the response sensitivity under high and low pressure, respectively. Moreover, the chain effect of BC and MXene nanosheets can increase interface stability and improve cycle performance (8000 compression-release cycles). The as-prepared pressure sensor features high sensitivity (528.87 kPa^−1^), a low detection limit (0.6 Pa), and a fast response speed (response time of 45 ms and recovery time of 29 ms). Zhao et al. [30] reported a multi-walled carbon nanotube/PDMS resistance strain sensor and pressure sensor. Using a simple and fast microstructure transfer method, a resistive pressure sensor with high sensitivity and wide linear range was fabricated by introducing a biomimetic spinous microstructure. A resistive pressure sensor can produce prominent and continuous dynamic responses under bending/restoring and stretching/releasing behavior (Figure 1e).

Liao et al. [31] coupled PDMS and PU and produced non-invasive, high-tensile sweat sensors. PDMS provides a flexible substrate and PU optimizes the adhesion between the electrode (Figure 1f) and the substrate, increasing the hydrophobicity of the electrode surface by introducing graphene–carbon nanotube materials. The sensor demonstrated a wide detection range of NH_4_^+^ from 10^−6^ M to 10^−1^ M with high stability and sensitivity, showing a high sensitivity of 59.6 ± 1.5 mV/log [NH_4_^+^] and an LOD lower than 10^−6^ M. Under a strain of 40%, the sensor still showed a sensitivity of 42.7 ± 3.1 mV/log [NH_4_^+^].

### 2.2. Thermosetting Polymers

Thermosetting polymers are formed by the irreversible curing of a viscous polymer. For example, the PI film is a stable material at high temperatures and strong acids and bases with high mechanical strength, which makes the processing and modification of PI compatible with more processes and is suitable for use as a base material for sensors [32,33,34]. However, PI is usually not colorless and does not recover under tremendous pressure, limiting its application in wearable, transparent, flexible sensors [35]. The advent of colorless polyimide (CPI) [36,37] has broadened the production of high-performance flexible sensors.

Moon et al. [38] developed bare amino acid-mediated cationic amphiphilic surfaces based on PI for wound healing and monitoring pH. Because PI surfaces are unsuitable for wound healing and have poor blood compatibility and low oxygen permeability. They modified the PI surface via a simple waterborne dipping process, which did not affect its physical properties. The modified PI surface can inhibit bacterial contamination through repulsion and simultaneously kill bacteria because it has cationic amphiphilic properties. The pH sensor was made with an Ag/AgCl reference electrode and polyaniline with Nafion resin deposited by screen printing. The entire device is covered with a modified PI film except for the sensor electrodes. PI, by incorporating different materials, is also a means of extending its scope of application. Kou et al. [39] mixed graphene quantum dots into PI to construct artificial synapses with high sensitivity and a wide corresponding range, which has a promising application in wearable bio-flexible sensors. Similarly, the combination of graphene and PI is also a means of preparing wearable sensors. Liu et al. [40] made a durable sensor by laser direct writing. In previous reports, lasers can directly induce PI tape into 3D porous graphene without any precursor [41]. Graphene is induced on PI by pulsed ultraviolet picosecond lasers. Digitally designed graphene patterns grown from the PI fabric surface follow the woven direction of the fiber, which demonstrates excellent sensitivity (GF_max_ = 27) in assembled strain sensors. Experimental results also showed that good linearity, low minimum strain response limit (strain = 0.08%), and good mechanical durability for over 1000 cycles can be achieved by using appropriate laser energy. In addition, real-time detection for finger and wrist bending and muscle tension proved the ability of the PI fabric strain sensors to monitor human-body activities.

### 2.3. Thermoplastic Polymers

Thermoplastic polymers can perform a reversible phase transition between solids and liquids [42]. Thermoplastic PU (TPU), one kind of thermoplastic material, has excellent elasticity, chemical stability, processing convenience, and cost-effectiveness [43,44,45]. TPU has a strong affinity with a variety of carbon and metal nanomaterials [46], and it has also been reported that cellulose nanocrystals (CNCs) are added to PU [47]. This combination of sensors combines the advantages of both, showing excellent tensile properties, a wide sensing range, excellent electrical conductivity, and high sensitivity [48]. In terms of microstructure design, the combination of a porous, cracked bionic structure, and the superior tensile properties of TPU can significantly improve the sensor’s sensitivity to a certain extent. The accuracy of the structural design can be a critical factor in improving the performance of sensors based on TPU substrates [49]. Vossmeyer et al. [50] combined a patternable crosslinked gold nanoparticle (GNP) film as an active conductive layer with a biodegradable PU film as a flexible substrate. A simple, clean, fast, scalable, and highly reliable contact printing method has been developed for transferring patterned GNP films to biodegradable PU films. The biodegradable PU film was prepared by casting, with a thickness of about 250 μm and a transmittance of about 92%. The GNP film is prepared on a slide through a layer-by-layer spin-coating procedure, where a sharp blade is used to scratch the GNP film on the glass substrate to obtain the desired size and shape. The PDMS impression then facilitates the transfer by touching the GNP surface. A small amount of water tunnel glass and GNP interface area are transferred to the PDMS impression by stripping the glass substrate. Finally, the PDMS stamp is placed on a biodegradable PU film, heated to 60 °C for 3 min, cooled to 0 °C for 3 min, and the GNP is transferred to the PU film. Combined with the soft PU substrate, the GNP-based sensor with a relatively low gauge factor of ≈101 enabled the accurate detection of subtle physiological signals, for example, measurements of well-resolved pulse waves and the action of laryngeal muscles during swallowing and speaking.

### 2.4. Liquid Crystalline Polymers

Liquid crystalline polymers can generate stable liquid crystal mesophase polymers under appropriate temperatures, pressures, and concentrations while having the properties of liquid flow and solid anisotropy. Liquid crystalline polymers can modulate the propagation of light in the external stimulation and have been widely used in the field of display, which also shows the potential to be an optical sensor, applied in ionic skin, photon skin, and electronic skin. Bai et al. [51] reported a study inspired by chameleon skin, presenting dual-sensing ironic skin (DSI-skin) based on electromechanical and mechanochromic material with multiple sensing functions, antibacterial properties, and antifreeze energy supply. This DSI-skin was fabricated by introducing Al^3+^ ions to offer ionic conductivity and highly substituted hydroxypropyl cellulose (HPC) to form cholesteric liquid-crystal structures in a poly (2-amino-4-pentenoic acid sodium-co-acrylamide) (PASCA) hydrogel scaffold. HPC is also reported by Hu et al. [52], who introduced additional photoelectric signal sensing capabilities to a double-network of ion conductive hydrogels with a high tensile strength, elongation, and toughness, greatly expanding this dual-mode flexible sensor’s important applications in writing recognition and electronic skin.

In addition, polymer-dispersed liquid crystal (PDLC) devices are also potential materials for intelligent electronic display applications, and such devices can be reversibly switched between transparent and opaque by voltage stimulation. For example, Zhang et al. [53] proposed self-assembled AgNWs micro nets. The stretchable transparent conductive electrodes (STCEs) were prepared by embedding them in a PDMS-flexible substrate. Based on this STCEs-assembled PDLC device with interactive pressure capabilities and stretchable capabilities, it can be used for sensing and light modulation. This kind of flexible liquid crystal polymer device has great application prospects in the field of wearable sensing healthcare devices.

### 2.5. Polymer Gels

Polymer gels, reported since 1978, are cross-linked polymer networks that swell in solvents that can respond to external environment change (pH, temperature, solvents, etc.) and perform a discontinuous and reversible change in the volume [54]. This feature inspired researchers to incorporate its response characteristics in wearable device application interests, such as releasing drugs [55], monitoring motion [56], and changing the tissue adhesion characteristics [57]. In addition, by combining gels and pressure-sensitive polymers (PVDF, PLA, etc.), strain sensors and pressure sensors with excellent performance can be fabricated.

Qin et al. [58] reported a hydrogel strain sensor with strong tensile strength (166 kPa), super-tensile properties (>1600%), and low delay in the detection of intense human activity and subtle physiological activity. It can be used as a bioelectrode for monitoring ECG and EMG. This hydrogel integrates hydroxypropyl methylcellulose (HPMC) and poly-(3,4-ethylenedioxythiophene): poly-(styrene sulfonic acid) (PEDOT: PSS) into a covalently cross-linked PAM network, which has good toughness and electrical conductivity, which shows potential applications in wearable healthcare electronic devices. Recently, it has also been pointed out that flexible electronic devices show difficulty in distinguishing ultra-small vibrations due to very low pressure, complex waveforms, and high noise sensitivity. To this end, Peng et al. [59] reported a sensor based on anisotropic conductive biphasic liquid metal-polymer gels that can detect more subtle vibration signals. The material is made by curing silicone polymers using conductive biphasic liquid metals (LMs) and insulating copper oxide particles with anisotropic conductivity. Even a small deformation can change the electrical contact of the particles and significantly affect the resistance to recognize extremely small signals with a high gauge factor (GF: 12,787 at strain and 4121 kPa^−1^ in stress). This strategy provides a new design idea for ultra-sensitive flexible electronic devices.

### 2.6. Intrinsically Conducting Polymers and Piezoelectric Polymers

Intrinsic conductive polymers (ICPs) have the electric, magnetic, and optical properties of metals and semiconductors [60] and are composed of conjugated sequences of double bonds or aromatic groups through redox conversion. The charge transfer complexes are formed by doping [61]. Common examples are polyacetylene (PA), polypyrrole (PPy), polyaniline (PANi), polythiophene (PTh), and PEDOT: PSS. ICPs also have other properties. For example, PEDOT and its derivatives, which have high transparency in the visible range, also expand potential applications of flexible transparent electrodes in wearable devices [62,63,64].

### 2.7. Biodegradable Materials

**Figure 2:**
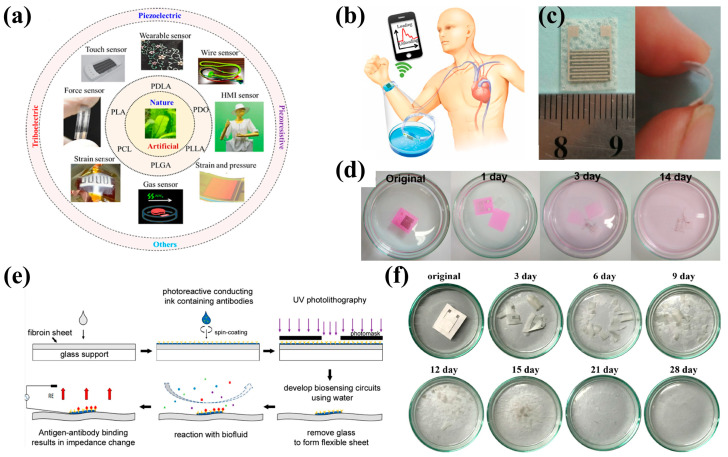
Wearable healthcare devices based on biodegradable material. (**a**) Biodegradable devices in various applications with various materials. Images reprinted from [68] with permission; (**b**) Schematic illustration of PLA/MXene sensor performance; (**c**) photograph of the flexible wearable transient pressure sensor; (**d**) biodegradable sensor’s performance in NaOH solution. Images reprinted from [71] with permission; (**e**) Microfabrication flow chart of the biosensor via photolithography of photo fibroin and sericin-based biocomposites. Images reprinted from [74] with permission; (**f**) MXene/protein-based biocomposites biodegradable performance in NaOH solution. Images reprinted from [73] with permission.

To avoid environmental pollution caused by large-scale use and to simplify the use of wearable devices, degradable materials have also received continuous attention from researchers [65]. Biodegradable materials have attracted extensive research interest due to their transparency, environmental friendliness, renewability, and piezoresistive effects. Many biodegradable materials have been reported in these years [66,67]. These materials include degradable polymers, semiconductors, and hydrolyzed metals, which can be partially or completely degraded under the appropriate pH, pressure, temperature, microorganisms, and other external environmental conditions. Natural polymers are common materials for biodegradable sensors, such as protein-based polymers (collagen, gelatin), and polysaccharides (starch, cellulose). In addition, synthetic biodegradable materials can be more customized for specific physical and chemical properties and mass production. These materials can be processed by electrospinning, 3D printing, and melt-spinning, etc. A variety of degradable materials (Figure 2a) can be used to build sensing and energy-harvesting wearable devices, including polylactic acid (PLA), polylactic glycolic acid (PLGA), material poly (L-lactic acid) (PLLA), poly-γ-glutamic acid (PGA), polycaprolactone (PCL) and so on [68]. Degradable polymers are usually degraded by cracking at unstable sites along the backbone of the polymer chain. These materials can be accelerated by biological enzymes, facilitated by reactive oxygen species (ROS) at the wound site, are dependent on ester bonds in the polymer to decompose in acid and base, temperature, and the physiological composition of body fluids to facilitate the degradation process. In addition, in addition to silicon nanomembranes, semiconductor materials have been reported for their degradable applications [69]. Mg, Mo, W, Fe, and other metal materials are also used in biodegradable sensors. The effects of oxidation products and decomposition products of these metal materials on the human body need to be considered [70]. In addition, triboelectric/piezoresistive, natural materials, etc., have significantly promoted the development of environmentally friendly wearable sensors. 

Guo et al. [71] reported a biodegradable sensor based on PLA sheets and porous MXene (Figure 2b,c). The flexible wearable transient pressure sensor is fabricated by impregnating MXene nanosheets into tissue papers (MXene/tissue paper) with a porous structure, recyclability, low cost, degradability, and reliable elasticity. A biodegradable PLA sheet and a PLA sheet patterned with an interdigitated conductive electrode are then sandwiched between them. The as-prepared wearable pressure sensor achieves high sensitivity, a low detection limit (10.2 Pa), ultrasensitive loading sensing of a sugar granule (2.3 mg), fast response (11 ms), low power consumption (10^–8^ W), and excellent reproducibility over 10,000 cycles and is biodegradable (Figure 2d).

Venkatarao et al. [72] reported a preparation method of a wearable device that writes graphene directly onto cellulose paper by preparing graphene particles into graphene pencils, which are directly written onto 120-micron thick paper to make a sensor. This sensor identifies different breathing patterns and rates by peak current and frequency. When measured after the individual drank water, the response increased by 25–35%, indicating that the individual’s water and levels could be monitored. Because graphene nanosheets can induce thermal radiation effects, a 34% enhancement in the fall time of the breath sensor was observed when infrared illumination was received. In addition, protein-based biodegradable composites (Figure 2e,f) are also one of the research hotspots [73,74].

### 2.8. Self-Healing Materials

Current wearable medical devices are often limited to substrates or electrodes. In the complex movement of the human body, it is easy to cause wear and even damage to the components. Even minor wear and tear can significantly shorten the life of a wearable sensor, resulting in a waste of resources and difficulty in controlling the life cycle. Therefore, a hot spot of research is in developing a wearable medical device like human skin with self-healing functions. In recent years, substrate materials with self-healing functions have grown rapidly, greatly extending the use time of wearable devices. 

The mechanism property of self-healing material can be divided into extrinsic and intrinsic [75]. Extrinsic self-healing materials usually contain microcapsules as well as vascular networks or other agents and structures that can be activated when the material is damaged. For example, when the material of the pre-embedded microcapsule is damaged, the capsule will break under the excitation of cracks, and by the capillary effect, release into the crack of the material to repair the crack [76], which can respond to various types of damage, and has a good healing efficiency. This method also has disadvantages: the possibility of leakage of the repair agent in the capsule and the concentration that directly affects the self-healing performance of the device and the repair efficiency of the capsule will be greatly reduced after multiple injuries. In addition, the self-healing vascular networks mimics the function of biological blood vessels. When the material is damaged, the vascular network at the corresponding position also breaks and the internal healing agent can be precisely released to achieve the purpose of self-healing the device. Compared to the microcapsules, the integrated nature of the vascular network makes it easier to refill, also with a greater repair area [77]. However, the impact of the presence of the vascular network on the structural integrity of the device needs to be considered and the complexity and cost of the process can also be a challenge. 

In contrast, intrinsic self-healing materials do not require external healing agents, which are reliant on dynamic covalent bonding reactions and non-covalent bonding interactions [78,79], such as Diels–Alder and retro-Diels–Alder reactions, trithiocarbonate reshuffling reactions, triazolinedione-based click, and dynamic urea bonds, as well as hydrogen bonds, metal-ligand bonds ionic interactions, π–π interactions, host–guest interactions, and Van der Waals interactions. Materials based on dynamic covalent bonds mostly require external stimuli to activate the healing process, such as light, heat, and electricity, etc. Supramolecular interactions based on non-covalent bonds can achieve self-healing. Such intrinsic materials can undergo multiple self-healing periods, reduce costs, and allow wearable devices to have a greater sustainable performance. This section will focus on self-healing wearable devices based on hydrogels and liquid metals.

**Figure 3:**
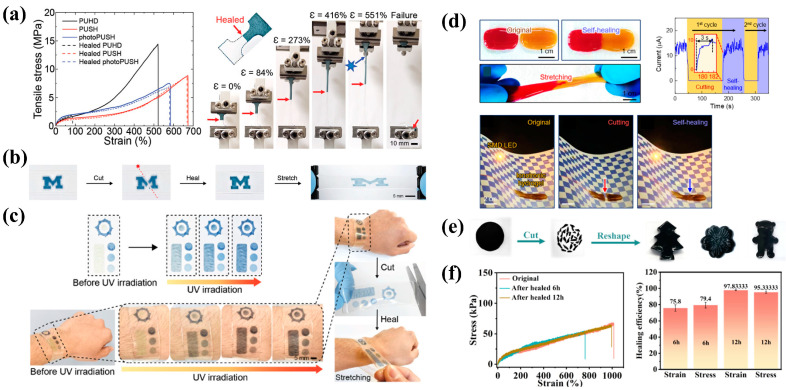
Self-healing wearable sensors based on hydrogels. (**a**) Tensile stress–strain curves of photoPUSH substrate sensors before and after healing; (**b**) “M” shape photoPUSH hydrogel was subsequently healed at 70 °C for 24 h; (**c**) photoPUSH-based UV sensor can perform normally after healing. Images reprinted from [80] with permission; (**d**) images of PPT hydrogels exhibiting rapid self-healing abilities. Images reprinted from [85] with permission; (**e**) PBM can be reshaped after cutting in pieces; (**f**) Stretch curve of PBM hydrogel after self-healing at different times and healing efficiency of PBM hydrogels after tensile fracture healing. Images reprinted from [87] with permission.

Yimyai et al. [80] proposed a method of incorporating phosphomolybdic acid (PMA) into a dynamic polyurethane polymer network with a reversible self-healing photochromic elastic composite (photoPUSH) of disulfide bonds (PUSH), which can change color by electron donor groups without an additional doping dose (Figure 3a,b), has good tensile properties and durability, and uses dynamic bonds to repair extreme mechanical damage. The adhesive properties also make it easy to integrate with other materials. Based on this material, the team developed an ultraviolet (UV) sensor sticker that can be attached to various surfaces and change from colorless to blue after eight hours of natural sunlight. At the same time, wearable wristbands with integrated UV-sensing patches have also been created, which can self-heal at 70 °C after undergoing extreme cutting (Figure 3c). In addition, the wristband can repair and sense in wet environments, which is not possible for many wristbands relying on electronics and water-sensitive substrate sensors.

Flexible conductive hydrogels are widely used in wearable devices [81,82,83,84]. By incorporating conductive polymers, ionic conductive materials, and fillers, the conductivity of hydrogels can be significantly increased. The combination of biocompatible materials can show low cytotoxicity and prolong wearing time. Seong et al. [85] report 3D printable, self-adhesive, self-healing, and conductive ionotropic hydrogels based on polyvinyl alcohol (PVA), pectin, tannic acid (TA), and borax, called PVA–pectin–tannic acid (PPT) hydrogels. The hydrogels exhibit strong adhesion to pig skin, gloves, glass, and plastic, excellent electrical conductivity exhibiting sufficient sensitivity [gauge factor (GF) = 2.5], and a wide sensing range (approximately 2000%). The complex pattern of self-healing (Figure 3d) can be printed directly on the flexible substrate, which can be used as a wearable strain sensor to monitor various human movement behaviors. The Cicoira group [86] also reported a self-healing conductive film, mixing poly(3,4-ethylenedioxythiophene) doped with polystyrene sulfonate (PEDOT: PSS), ethylene glycol (EG), and TA preparation, has a strong adhesion on a variety of substrates. This film is non-cytotoxic, and the epidermal electrodes of the vegetation of these materials have low skin-electrode impedance at low frequencies (1–100 Hz) and have high-quality electrocardiogram (ECG) and electromyogram (EMG) signal recording. Similarly, Wang et al. [84] reported wearable devices, based on the hydrogel network, that can detect tiny body movements and gestures and identify different handwriting. More recently, Sun et al. [87] integrated MXene nanosheets into a hydrogel (PBM hydrogel) that utilizes dynamic reversible borax ester bonds and multiple hydrogen bonds between the components to achieve up to 97.8% self-healing efficiency and strong self-adhesive capabilities (Figure 3e,f). The skin sensor based on this hydrogel has a fast response time (10 ms) and can detect various human movement signals.

**Figure 4:**
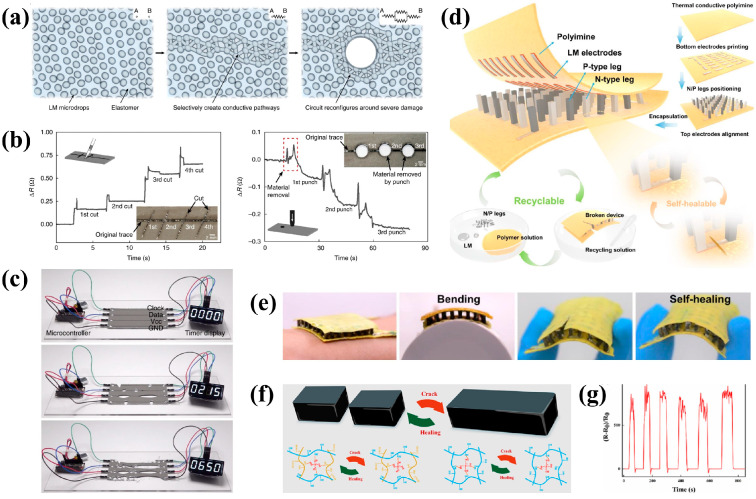
Liquid-metal-based wearable healthcare devices. (**a**) schematic illustration of the self-healing mechanism following damage, the LM trace autonomously reconfigures and maintains electrical conductivity; (**b**) resistance as a function of time for high-aspect-ratio damage from a razor blade shows that the circuit is autonomously reconfigured without intervention or loss of conductivity; (**c**) when severe damage is induced, the counter maintains operation, which requires all four traces to constantly maintain electrical conductivity. Images reprinted from [101] with permission; (**d**) the figure shows the principle of the device fabrication process and healing properties; (**e**) photos of a broken device before and after self-healing. Images reprinted from [102] with permission; (**f**) principle of self-healing of LMCNF-2 hydrogel; (**g**) real-time detection of resistance of the hydrogel over several cut and self-healing processes. Images reprinted from [103] with permission.

In addition, liquid metal is a very promising material for medical applications. It is widely used in flexible devices based on the manufacturing, patterning, and LM particle composites [88,89]. In addition to its inherent ability to conduct electricity, liquid metal has the properties of both liquid and metal, which makes it widely used in stretchable flexible electronics [90,91,92]. Wearable functional devices often use metal patterns to achieve various properties. Liquid metal is easier to process into patterns. Also, due to its flow characteristics, it is possible to control the distribution of liquid metal in the device and self-healing of the metal pattern or metal network. LM can be modified to improve its application range, such as electromechanical functionalization, sintering [93,94,95], thermal functionalization [96,97], biochemical functionalization [83,98], electromagnetic functionalization [99], magnetic functionalization [83,100], and self-healable functionalization [101]. To obtain self-healing properties, there are two main methods: exogenous self-healing methods, and the combination of healing agents based on microcapsules or microvessels ; and intrinsic self-healing methods, which rely on dynamic reversible covalent or non-covalent bonds to repair damaged areas (Figure 4a–c) [101]. Recently, Mao et al. [102] reported a self-healing and recyclable wearable thermoelectric device (Figure 4d,e). The device is integrated from dynamic covalent thermoset polyimide, LM, and thermoelectric legs to develop a self-healing and recyclable flexible thermoelectric device (f-TEDs) that achieves a standardized power density of 1.54 μW·cm^−2^·K^−2^. with a high coefficient of performance (COP) at 3.91 under 7 °C temperature difference, which leads to a low power consumption of ~38 W for the cooling of regular human body. It provides more application prospects for developing a wearable energy collection and personal thermal management. 

The combination of flexible hydrogel and LM is also a novel preparation idea. Feng et al. [103] reported a novel method based on constructing a double cross-linked network cellulose-based LM hydrogel (LMCNF) (Figure 4f,g) based on hydrogen and hydrogen dynamic boronic ester bonds through the introduction of LM, achieving a good antibacterial performance and EMI shielding performance. This sensor exhibits self-healing properties due to the dynamic hydroxy–borate network, and the tensile properties can be recovered after 60 min of self-healing; LM realizes the material’s high electrical conductivity (22.08 S/m), good EMI shielding (23.86 dB) and antibacterial properties, which have great application potential in wearable devices.

## 3. Applications

**Figure 5:**
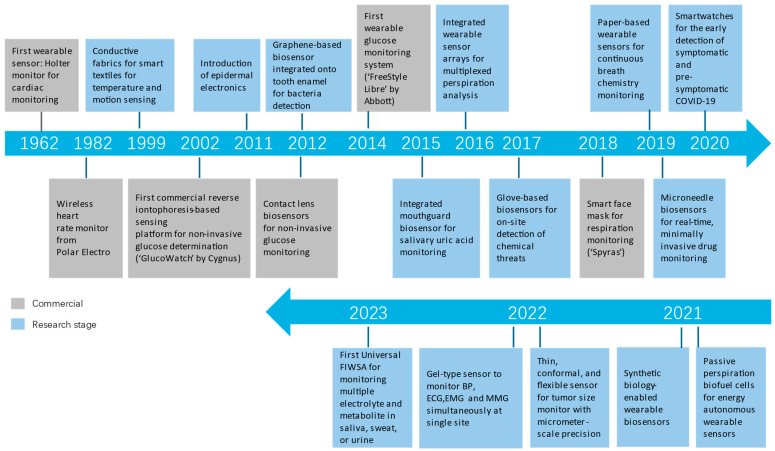
Timeline of significant milestones in the development of wearable sensors. (This diagram is based on a figure in [111]. Images reprinted with permission.) Added representative works in 2022 [112,113] and 2023 [114].

Different body parameters reveal health status, diseases, and sub-health. Wearable biosensors can collect an extensive range of data in real time for a long time, which enables continuous health monitoring [104], early warning [105,106,107,108,109], and disease screening [110]. Figure 5 shows the significant improvement timeline. Wearable sensors have developed to self-power, work in real-time, and possess multiplex sensing.

The first wearable sensors are based on a variety of physical mechanisms (called first-generation), such as temperature, pressure, stress, strain, piezo resistance, and piezoelectric effect, etc. Afterward, wearable sensors based on biofluids (called second-generation) appear. In this section, we will provide some representative examples of physical-based sensors applications, and then focus on the applications of biochemical-based sensors. 

### 3.1. First Generation: Physical-Based

**Figure 6:**
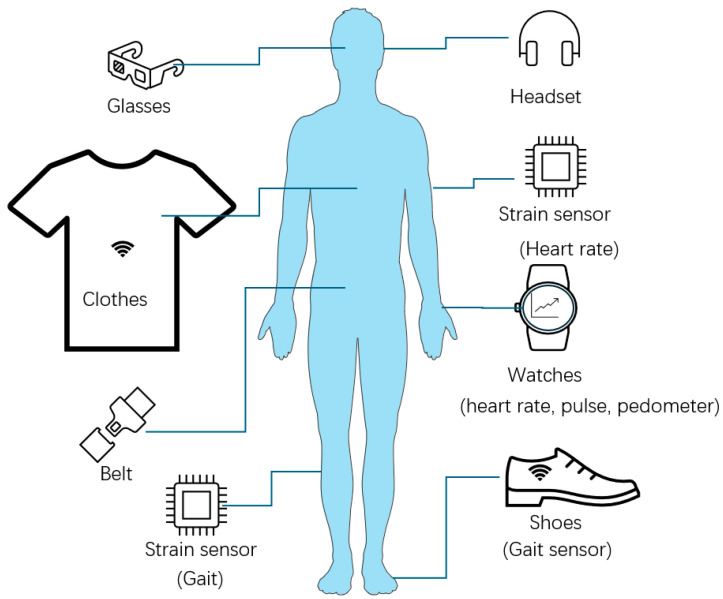
First-generation wearable biosensors examples (This diagram is modified from a figure in [117]. Images reprinted with permission).

The first-generation wearable biosensor devices are based on physical mechanisms and can be smartphones, smart watches, shoes, or headphones [111]. Figure 6 shows some typical examples. Among them, smartwatches have been widely accepted. Many applications, such as pedometers on smartwatches or smartphones, are designed for travel history, fitness, and exercise [111,115,116]. Travel history is widely used during the coronavirus disease (COVID-19) pandemic [115]. 

Some sensors are used to monitor vital signs [118], including respiration [119], temperature [120,121,122,123], blood pressure [124,125], heart rate, and pulse [126,127,128,129,130]. For example, wearable piezoresistive sensors can monitor the pulse and electrocardiograph accurately, even during running [131,132]. For a healthy adult, a normal respiration rate is typically between 12–20 breaths per minute (bpm) . The rate can reach 60 bpm during high-intensity exercise. Wearable sensors supply real-time monitoring and diagnosis, which may help patients with respiratory diseases, such as sleep apnea, asthma, and chronic obstructive pulmonary disease (COPD) [119].

Some sensors focus on gait analysis for fracture healing. Wearable sensors can continuously monitor the gait status and reach the healing process [133,134]. They can also be applied to some diseases, such as Parkinson’s disease [135]. Most are stress, strain sensors, and IMUs attached to the patient’s leg, shoes, or bottom of their foot [136,137,138].

In addition to physical health, wearable biosensors are also used in human psychological health. EEG, skin conductance (SC), ECG, and EMG are common stress detection methods [139,140]. Waleed uses heart rate, skin temperature, breathing rate, and skin conductance to monitor construction workers’ physical and mental stress and achieve an accuracy of 94.7% [141]. Florian et al. [142] reported that heart-rate variability (HRV) and breathing-rate variability (BRV) change a lot when the non-anxiety-induced and anxiety-induced states change. There are so many types of physical-based wearable sensors. Here, we mainly focus on bioelectrical sensors (EEG, ECG, EMG), and inertial measurement units (IMU).

#### 3.1.1. Bioelectrical Sensors

The human body can generate bioelectricity, and it can be collected to record and trace the bioelectrical activity of neurons, which reflects body status. EEG, ECG, and EMG are three types of medical techniques used to record the electrical activity of the brain, heart, and muscles, respectively. 

EEG first appeared in 1924, and now EEG examination is popularized in global hospitals as a mature technology. It is currently the most sensitive method to reflect the status of the brain [143]. EEG has been widely used to monitor the status (brain death [144], sleep abnormalities [145], etc.) of severe patients [146,147,148] and can save many lives by monitoring epileptic seizures, status epilepticus, and acute ischemic stroke in real-time [146,149]. Zhuang et al. reported spinal cord stimulation may facilitate the recovery of consciousness, and they used an EEG test to predict the process [150]. Thanks to the boom in deep learning, many deep learning models are developed and combined with EEG to detect mental fatigue [151], Parkinson’s disease [152], depression [153], schizophrenia [154,155], epilepsy [156,157,158], and neurocognitive disorders [159,160]. In addition to symptom detection, EEG is reported to localize the epileptogenic zone [161,162] and it has the potential to guide the surgery. Kucikiene et al. [163] found different EEG features of focal epilepsy and psychogenic nonepileptic seizures. In recent years, the braincomputer interface (BCI) has become a hot spot, and EEG is a core aspect. BCI provides a novel humanmachine interface (HMI) to control artificial limbs or external assistive devices [164].

In 1887, Waller first introduced the ECG. In 1903, Einthoven invented the first practical ECG device and was awarded the Nobel Prize for this achievement. ECG has become the core of critical conditions diagnoses like ventricular tachycardia, atrial fibrillation, and acute myocardial infarction [165]. The advent of wearable ECGs has enabled long-term monitoring of ECG time variations, leading to the development of dynamic ECG. Recently, Pham et al. presented that dynamic ECG changes can be a new risk marker of sudden cardiac death [166]. Similar to EEG, with the recent explosive development of AI, combining AI with ECG is promising. Soh et al. used machine learning and energy waveform electrocardiogram to detect subclinical left ventricular dysfunction [167]. Zhang et al. used an information bottleneck-based multi-scale network to detect ECG arrhythmia [168]. Lee et al. applied AI and ECG to measure filling pressure and left ventricular diastolic function [169]. Chen et al. used deep learning and ECG to detect pediatric congenital heart disease [170].

EMG can monitor muscle status for sports rehabilitation, HMI, giant analysis, and stroke rehabilitation [171,172,173,174]. Park et al. [175] developed reusable dermal surface EMG to control lower extremity robotic legs. Its serpentine electrode pattern enhances its effective contact area, durability, stretchability, and signal-to-noise ratio (SNR). Chand et al. used surface EMG dynamic muscle fatigue assessment [172]. Li et al. presented a 3D-printed, flexible, and stretchable smart textile for ECG and EMG [176]. 

#### 3.1.2. IMU

IMUs are sensors based on inertia and relevant measuring mechanisms. Two typical IMUs, accelerometers, and gyroscopes, are used to measure specific forces and rotations [177]. Micro-electromechanical-systems (MEMS) technology makes IMUs portable and wearable [178]. Wearable IMUs are widely used in gait parameters [179,180], Parkinsonian and seizure attack detection [181], monitoring exercise [181], postural feedback [182], human movements analysis [183], HMI [184], and so on. 

IMUs are widely used for HMI and feedback control systems. Dey and Schilling [185] proposed a prediction network based on IMU to predict foot angle trajectory in real time, offering a possible architecture control of powered intelligent prostheses. Peng [186] developed an aerial continuum manipulator using IMU. Its closed-loop kinematic controller enhanced robustness. Zhang et al. [187] used exosuits based on IMU to reduce users’ energy consumption by 14%. 

Additionally, wearable IMUs are used to monitor the safety behaviors of scaffolding workers [188], enhance human location with Wi-Fi sensing [189,190], count exercise repetition [191], and assessment of knee moments [192]. More applications of IMUs can be found in these reviews [193,194,195,196,197,198,199].

### 3.2. Second Generation: Biochemical-Based

**Figure 7:**
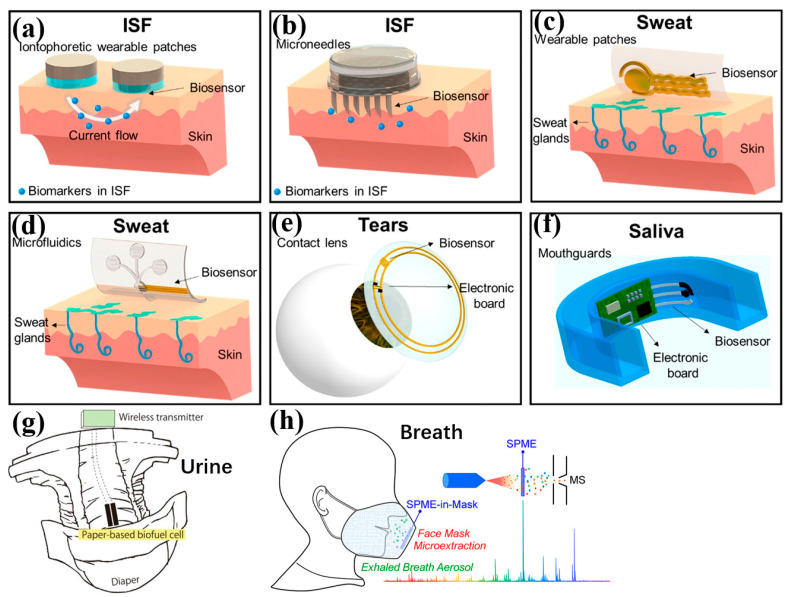
Schematic diagram of the biosensors using different body fluids: (**a**) and (**b**) ISF, (**c**,**d**) sweat, (**e**) tears, (**f**) saliva, (**g**) urine, and (**h**) breath. Images reprinted from [210,234,235] with permission.

Serum and blood plasma have been the gold standard for diagnosis for a long time [200]. However, taking blood is invasive and painful, thus, it’s unsuitable for real-time, point-of-care diagnosis. Therefore, people desire alternative solutions with other body fluids, such as saliva, sweat, tears, and interstitial fluid (ISF). Wearable biosensors using these body fluids belong to the second generation. Table 1 lists their features and comparisons. Figure 7 shows their schematic diagram. The biomarkers in these biofluids can reveal the human body’s status. A variety of active materials can detect the substances in biofluids. Biomarkers are usually converted to photosignals or electrical signals by some active materials, like micro/mano-structured bioelectronic devices(M/NBDs), surface-enhanced Raman spectroscopy (SERS), molecularly imprinted polymers (MIPs), biofluidic material-based carriers (BMCs), MXene, aggregation-induced emissions (AIEs) bioprobes, and metal-organic frameworks (MOFs) [116,201,202,203,204,205,206,207,208,209]. For example, enzymes can convert metabolites to other detectable substances or directly convert them to electrical signals [210].

**Table 1:** Comparison and characteristics of biofluids or breath [111].

Sample	Target Biomarkers	Wearable Format	Diagnostic Examples	Advantages	Disadvantages
**ISF**	Metabolites, ions, circulating RNAs, proteins, amino acids, fatty acids, peptides, coenzymes, neurotransmitters, hormones [111,116,200,211]	On-skin patch	Metabolite detection: glucose, lactate, ketone bodies, alcohol and uric acid PH sensing [212] Neurotransmitter detection Drug monitoring [213]	Rich source of Biomarkers [214,215,216] Minimally invasive Location (near the skin surface) Similar composition with blood plasma, serums Stead and continuous secretion rate Skin offers a large interface	Invasive Discomfort from sampling approaches. The time lag between interstitial and blood analyte levels. Low sample volume for analysis Skin thickness variation between individuals and sites
**Sweat**	Metabolites, electrolytes, irons, proteins, peptides, neurotransmitters, fatty acids, hormones [111,214,215,216]	On-skin patch, tattoos, clothes	Metabolite detection: glucose, lactate, alcohol and uric acid Protein biomarker detection: TNF Chronic disease monitoring: inflammatory bowel disease, cystic fibrosis [217] pH sensing [218,219,220] Hormone detection: cortisol	Convenient Non-invasive Location (on the skin surface) Skin offers a large interface Sample continuously secreted Sweat glands are widely distributed	Low volumes at normal sweat rates Contamination Evaporative loss Dilute analyte concentrations Compositional variation depending on the area of sampling Variation in sweating rates
**Breath**	Metabolites (volatilized or in aerosols), VOCs, viruses [111,221,222,223]	Face mask, Electronic nose	Metabolite detection: H_2_O_2_, SARS-CoV-2 testing	Non-invasive Convenient Sample continuously generated	Limited biomarkers, except for VOCs Face masks might be uncomfortable for users. VOC detection would require notable sensor engineering Unique sampling requirements for aerosol capture
**Tear** **s**	Metabolites, electrolytes, proteins, hormones, lipids [111,224,225,226]	Contact lens	Metabolite detection: glucose and lactate	Convenient Non-invasive Sample continuously secreted	Lag between tear and blood analyte levels. Eye position requires considerable device engineering. The correlation between blood and tear analyte might be weak
**Saliva**	Metabolites, electrolytes, proteins, hormones, bacteria, and viruses [111,227,228,229]	Mouth-guard, on-tooth patch, pacifier [230,231]	Metabolite detection: glucose, lactate, alcohol and uric acid pH sensing [220] Specific bacterial monitoring Drug and hormone testing	Convenient Non-invasive Sample continuously secreted	High viscosity might pose sampling problems. Variation in analyte correlation between blood and saliva Saliva production changes due to talking, eating, or drinking. Eating or drinking brings contamination. Difficult for comfortable long-term use
**Urine**	Metabolites, electrolytes, metals, peptides, amino acids, fatty acids, toxins, hormones, proteins, coenzymes, neurotransmitters, circulating RNA and DNA [111,232,233]	Diaper [234]	Metabolite detection: glucose, nitrate pH sensing [220]	Rich source of biomarkers Convenient Non-invasive	Rely on urination events

ISF, interstitial fluid; SARS-CoV-2, severe acute respiratory syndrome coronavirus 2; VOC, volatile organic compound.

#### 3.2.1. Interstitial Fluid

Interstitial fluid (ISF) can be seen as a cell-free part of blood plasma. Moreover, some disease biomarkers might appear in ISF but are absent in blood. Therefore, ISF provides a rich source of biomarkers [111]. Compared to blood, ISF does not coagulate [213]. In addition, taking ISF is much less invasive and less painful than taking blood, which is a great advantage, especially during long-term monitoring. In addition, suction blisters, microdialysis, sonophoresis, and thermal ablation are also reported [236].

Currently, the main methods of ISF sampling are iontophoretic extraction and microneedles. Both have been developed over decades and are relatively mature. Iontophoresis extraction uses a low-voltage electric current in a skin region, leading to the electromotive migration of charged molecules. Microneedles were initially developed for drug delivery and later became a common approach for minimally invasive biofluid sampling in wearables. They aim to puncture through the stratum corneum and epidermis to access the dermis. Microneedles usually contain a single or an array of microscopic structures fabricated from hydrogels or biocompatible synthetic polymers [111]. Abbasiasl et al. [237] developed fully integrated touch-activated hollow microneedles for ISF continuous sampling and sensing. The platform’s strength is that it is fully integrated and the operation is super simple (just one touch).

Continuous glucose monitoring (CGM) allows painless, continuous, minimally invasive long-term glucose monitoring and has become the new standard of care for diabetic patients. Commercial CGM mainly depends on ISF. CGM has been combined with an insulin pump to complete a close-loop management for insulin and glucose. The Food and Drug Administration (FDA) of the U.S. has granted some related commercial products.

#### 3.2.2. Sweat

Perspiration helps people maintain thermal homeostasis and excrete chemicals and metabolites. Sweat has a lot of electrolytes, metabolites, and other biomarkers that link closely to health. In the past, researchers collected sweat with pads and analyzed it with benchtop devices. Wearable sweat sensors emerging in recent years have enlarged the research value of sweat because they can continuously collect various body statuses and parameters for real-time/long-period monitoring. Compared to ISF, taking sweat is less painful, more accessible, and non-invasive.

Hydration status monitoring is an essential application of wearable sweat sensors. Insufficient consumption of water causes dehydration, which can bring fatigue, headache, dizziness, and even life-threatening disease events like stroke. The concentration of Na^+^ and K^+^, can be used for monitoring the whole body’s sweat rate [214,238,239], which would be helpful for athletes and workers who work outdoors during summer.

Electric fiber is one of the most popular materials for wearable sensors. They are usually added to clothing, which requires good flexibility, strength, wearability, and permeability. Hekmat et al. [240] introduced binary nickel-cobalt to commercial cotton fabrics for glucose sensing. It has a wide linear concentration range (0.04–8.3 mM), quick response time (4.2 s), and small minimum detection concentration (0.116 μM). Mo et al. [241] developed a fabric sensor to measure K^+^. It has a 2.1 s response time over 100 min stability. Zhang et al. [242] developed a fabric wearable sensor for detecting real-time Na^+^, pH, and glucose. It is all made of fibers; therefore, it has excellent flexibility and comfortable wearability. In addition, it realizes highly efficient sweat collection during the monitoring.

However, the correlation between blood sweat and many parameters remains controversial. Some papers reported a good correlation, but others were contrary [210]. Bad correlation may result from the collection method, contamination, or inappropriate biomarker chosen.

#### 3.2.3. Saliva

Saliva contains ions, enzymes, proteins, metabolites, microorganisms, and hormones, some of which have been used clinically [243]—for example, a mouthguard for salivary uric acid biosensing (Figure 7f). Saliva has great potential to monitor glucose levels [231]. The study presented in [244] compared six saliva collection methods and found that unstimulated parotid achieves the highest glucose level and best correlation with blood glucose levels. It is non-invasive and easy to collect compared to finger prick tests and ISF.

For example, cortisol is a steroid hormone related to stress levels and some pathologies, such as Cushing’s syndrome and Addison’s disease. A directly proportional correlation is found between the circadian variations of cortisol concentration in saliva and blood, leading to several biosensors for cortisol salivary levels [245].

#### 3.2.4. Breath

Breath has been focused on in the first generation of wearable biosensors, but the composition of breath is not involved. Each breath contains a distribution of aerosols of different sizes. Activities such as coughing, sneezing, or talking can increase these breath aerosols a lot. These aerosols have a lot of biomarkers for respiratory pathogens, especially for diseases of the respiratory system. They can be collected with face mask integrated sensors, proven by COVID-19 viral nucleic acid [246].

Human breath contains tiny amounts of volatile organic compounds (VOCs), including nitrogen oxides, ammonia, hydrogen sulfide, acetone, methanol, pentane, isoprene, methane, ethanol, and ethane. These VOCs can be outsourced or endogenic. Outsourcing ingredients comes from environmental exposure, while endogenic ingredients are generated through various metabolic pathways. Therefore, abnormal VOC composition can indicate our poor health status, respiratory disease, and exposure to a seriously polluted environment [247,248,249].

Li et al. [250] developed an E-Nose to test COVID-19, reaching 79% correction. This system uses 64 chemical sensing elements and machine learning for judgment. The correction of the whole system boosts enormously compared to a single sensing element (repeatability is 0.02%, and reproducibility is 1.2%). In addition, E-Noses are applied to detect early lung cancer [251,252,253,254].

#### 3.2.5. Tears

Tear fluid is an important part of the eyes, responsible for lubricating, cleansing, and refractory purposes [255]. Tear fluid contains some biomarkers for eye disease [256]. For example, ascorbic acid levels in tears are closely related to ocular inflammation [257]. Contact lenses are a typical visual prosthetic device with over 150 million users worldwide. Due to their non-irritant property and popularity, most tear sensors are integrated into contact lenses to collect tears. Parviz et al. developed the first glucose sensors based on contact lenses in 2011 [258].

However, the correlation between blood and tear analyte might be weak. Additionally, contact with the eyes requires significant caution because eyeballs are sensitive. Due to the reasons above, it seems hard to compete with ISF, except for eye diseases. The potential for eye diseases cannot be ignored because monitoring for a long period is meaningful.

Li et al. [259] designed a power-free contact lens sensor for glucose sensing. Compared to former studies, it does not need neither peripheral nor power to transfer data. Instead, it uses the color change to Prussian blue to show the results. Its detection concentration ranges from 0.05 mM to 0.9 mM, with a correlation coefficient r = 0.99543 to the controlled group. Shi et al. [257] developed a contact lens sensor to monitor ocular inflammation. Its operational and storage lifetime reaches 20 h and 10 days, respectively. The BSA-Au nanocluster probe measures the biomarker ascorbic acid.

#### 3.2.6. Urine

Urine contains a lot of analytes, such as DAN, RNA, metabolites, and proteins. Due to the biomarkers in urine, urine tests are widely used in hospitals for urinary lithiasis, urinary tract infections, kidney disease, urinary tract infections, diabetes, liver problems, and genitourinary cancers [260,261,262,263,264,265,266]. Urinalysis can be divided into urine sediment analysis, urine culture, and urine chemical analysis. Due to the simple and non-invasive sampling process, many kinds of urine test strips were invented and widely applied for screening. Urine test strips are cheap and convenient with an acceptable accuracy decline, and can be used at home; therefore, they are widely used for commercial rapid pregnancy testing, urinary tract infections screening [262,267], diabetes management [261], and liver/kidney function monitoring [261,267,268]. In a word, urine is a promising biofluid for healthcare.

As the development of wireless technology and flexible electronics developed, urine wearable sensors appeared. The urine sensor depends on urination events [82], so it does not need to be wearable in most scenarios. Urine test strips especially are convenient enough. However, wearable urine sensors are significant for people who cannot express themselves or have urinary incontinence, such as babies and patients in a vegetative state.

Urine wearable sensors are usually integrated into diapers. Early urine sensors were based on temperature/humidity/conductivity to detect urination events and alert caregivers to change diapers. These sensors improve the life quality of the care recipient and its caregivers. Exposure to a soiled diaper for a long time can cause skin allergies and damage. Checking diapers frequently is time-consuming and may bring mental pressure to adult care recipients [269,270,271].

Seo et al. developed a self-powered sensor for urinary tract infection monitoring [272] in 2017. Shitanda et al. developed a diaper sensor for urine glucose [234]. Recently, multiplex sensing aroused people’s interest. Li et al. [273] and Yanni et al. [114] developed sensors to simultaneously measure multiple urinary metabolites and electrolytes based on a sensor array. The sensor reported in the study presented in [114] is the first universal fully integrated wearable sensor arrays (FIWSAs). It simultaneously supports multiple electrolyte and metabolite monitoring in saliva, sweat, and urine.

## 4. Future Perspectives and Conclusions

In this review, we mainly focused on the material and application of wearable healthcare devices. We introduced and discussed the substrate material with flexible, stretchable, biodegradable, self-healing properties used in healthcare devices. Wearable healthcare devices have become increasingly important in medical diagnosis and treatment. By using this non-invasive method, we can monitor interstitial respiration, temperature, blood pressure, heart rate, pulse fluid, sweat, saliva, breath, tears, and urine to obtain medical data directly or indirectly. The development of wearable healthcare devices has lasted for several decades.

At present, materials for wearable healthcare devices are the hotspot of research. The main purpose is to improve device sensitivity, cycle stability, usage life, environmental safety, broaden detection ranges, and advance applications. Most of these studies focus on a variety of new materials and optimizations based on existing materials including MXene [274], MOF [131,275], and graphene [276], etc. For example, in the case of graphene and ultra-thin electrodes, although they have excellent electrical properties, they are easy to damage, and the combination of additional flexible protective materials will increase the weight of the wearable instrument, and the thickness which can affect the user’s experience. Designing the microstructure of the material is also a method to improve the performance of the sensor, but the stability of the microstructure under the long-term wear of the human body, the influence of body fluids, and intense exercise also need to be taken into account. In addition, due to cost and other issues, research of novel characteristics, such as self-healing and biodegradation, has mostly stayed in the laboratory stage. The development of processes with high-volume production and low costs also needs to be the focus of future research.

In addition, there are still several challenges not limited to materials the future needs to solve.

### 4.1. Data Reliability

Ensuring the monitored data accuracy and reliability is the primary challenge. Devices may perform differently on a person with different behaviors or under different environments. How to adapt to a measurement environment affected by multiple factors is critical to the acceptance of wearable devices.

### 4.2. Data Privacy

The collection of health data by wearable devices, given its sensitivity, significantly raises concerns regarding privacy and security. Much data obtained by the device can be uploaded to the cloud. It is vital to safeguard this data against unauthorized access and breaches. The key challenge lies in deploying strong encryption techniques and data protection measures that do not detract from the device’s efficiency or the user’s experience.

### 4.3. User-Friendly

User-friendly devices are easier for patients to accept. Compared to hospitalization, personal wearability lacks the supervision of the doctor. Wearable technology of the future will seamlessly merge with everyday items like clothing, accessories, and implants. This integration will enhance user adherence and pave the way for new possibilities in constant health tracking without requiring active participation from users.

### 4.4. Battery Harvesting and Storage

The battery provides power to wearable devices, which guarantees the basic performance of devices. At present, the battery capacity of wearable devices is low, easy to damage, and sensitive to environmental changes [277]. The development of batteries with self-powered, flexible, biocompatible, breathable, moisture resistant, and other characteristics can greatly extend the life and application range of wearable healthcare devices. In addition, progress in energy harvesting methods [278], which transform body heat or motion into electrical power, may overcome the constraints of battery life. Such developments would allow devices to function for extended periods or potentially without end, negating the need for recharging and boosting their effectiveness for ongoing monitoring and reducing resource waste.

### 4.5. Healthcare System Integration between Devices and Medical Professionals

Merging data from wearable devices with the current healthcare system while making sure data are useful for medical professionals presents a complicated hurdle. To achieve a smooth exchange and understanding of data across various platforms and systems, it is essential to establish and implement standards and protocols for interoperability.

The development of new sensor technologies is expected to improve the accuracy and scope of health indicators monitored by wearable devices. Innovations in nanotechnology, flexible electronics, and biocompatible materials will enable more health monitoring capabilities, including real-time disease detection and monitoring. With the rise and rapid development of artificial intelligence, it is expected to accelerate the analysis and personalized evaluation of devices. While wearable medical devices face significant challenges, the future holds great promise in transforming healthcare delivery and management. Advances in technology and a focus on user-centered design are critical to overcoming these barriers and realizing the full potential of wearable medical technology. This new detection technology can significantly help doctors achieve early diagnosis, prevent diseases, and greatly reduce medical costs.

## References

[1] Shen G. (2021). Recent advances of flexible sensors for biomedical applications. Prog. Nat. Sci. Mater. Int..

[2] Pang C., Lee C., Suh K.-Y. (2013). Recent advances in flexible sensors for wearable and implantable devices. J. Appl. Polym. Sci..

[3] Iqbal S.M.A., Mahgoub I., Du E., Leavitt M.A., Asghar W. (2021). Advances in healthcare wearable devices. NPJ Flex. Electron..

[4] Updike S.J., Hicks G.P. (1967). The Enzyme Electrode. Nature.

[5] Pillai S., Upadhyay A., Sayson D., Nguyen B.H., Tran S.D. (2022). Advances in Medical Wearable Biosensors: Design, Fabrication and Materials Strategies in Healthcare Monitoring. Molecules.

[6] Nasiri S., Khosravani M.R. (2020). Progress and challenges in fabrication of wearable sensors for health monitoring. Sens. Actuators A Phys..

[7] Windmiller J.R., Wang J. (2013). Wearable Electrochemical Sensors and Biosensors: A Review. Electroanalysis.

[8] Liu Y., Wang H., Zhao W., Zhang M., Qin H., Xie Y. (2018). Flexible, Stretchable Sensors for Wearable Health Monitoring: Sensing Mechanisms, Materials, Fabrication Strategies and Features. Sensors.

[9] Liu J., Liu M., Bai Y., Zhang J., Liu H., Zhu W. (2020). Recent Progress in Flexible Wearable Sensors for Vital Sign Monitoring. Sensors.

[10] Windmiller J.R., Bandodkar A.J., Parkhomovsky S., Wang J. (2012). Stamp transfer electrodes for electrochemical sensing on non-planar and oversized surfaces. Analyst.

[11] Chan B.-D., Hsieh K.-H., Yang S.-Y. (2009). Fabrication of organic flexible electrodes using transfer stamping process. Microelectron. Eng..

[12] Li Q., Luo S., Wang Y., Wang Q.-M. (2019). Carbon based polyimide nanocomposites thin film strain sensors fabricated by ink-jet printing method. Sens. Actuators A Phys..

[13] Bariya M., Shahpar Z., Park H., Sun J., Jung Y., Gao W., Nyein H.Y.Y., Liaw T.S., Tai L.-C., Ngo Q.P. (2018). Roll-to-Roll Gravure Printed Electrochemical Sensors for Wearable and Medical Devices. ACS Nano.

[14] Hubble L.J., Wang J. (2019). Sensing at Your Fingertips: Glove-based Wearable Chemical Sensors. Electroanalysis.

[15] Zhuo Y., Hu H., Wang Y., Marin T., Lu M. (2019). Photonic crystal slab biosensors fabricated with helium ion lithography (HIL). Sens. Actuators A Phys..

[16] Han T., Kundu S., Nag A., Xu Y. (2019). 3D Printed Sensors for Biomedical Applications: A Review. Sensors.

[17] Gao M., Li L., Song Y. (2017). Inkjet printing wearable electronic devices. J. Mater. Chem. C.

[18] Lou Z., Wang L., Jiang K., Wei Z., Shen G. (2020). Reviews of wearable healthcare systems: Materials, devices and system integration. Mater. Sci. Eng. R Rep..

[19] Kim S., Kang J., Lee I., Jang J., Park C.B., Lee W., Bae B.-S. (2023). An intrinsically stretchable multi-biochemical sensor for sweat analysis using photo-patternable ecoflex. npj Flex. Electron..

[20] del Bosque A., Sánchez-Romate X.X.F., Calvo D.L.L., Fernández P.R., Borromeo S., Sánchez M., Ureña A. (2023). Highly Flexible Strain Sensors Based on CNT-Reinforced Ecoflex Silicone Rubber for Wireless Facemask Breathing Monitoring via Bluetooth. ACS Appl. Polym. Mater..

[21] Zheng Q., Jia C., Sun F., Zhang M., Wen Y., Xie Z., Wang J., Liu B., Mao Y., Zhao C. (2023). Ecoflex Flexible Array of Triboelectric Nanogenerators for Gait Monitoring Alarm Warning Applications. Electronics.

[22] Zhang S., Rana S.S., Bhatta T., Pradhan G.B., Sharma S., Song H., Jeong S., Park J.Y. (2023). 3D printed smart glove with pyramidal MXene/Ecoflex composite-based toroidal triboelectric nanogenerators for wearable human-machine interaction applications. Nano Energy.

[23] Liu G., Lv Z., Batool S., Li M., Zhao P., Guo L., Wang Y., Zhou Y., Han S. (2023). Biocompatible Material-Based Flexible Biosensors: From Materials Design to Wearable/Implantable Devices and Integrated Sensing Systems. Small.

[24] Lee P., Lee J., Lee H., Yeo J., Hong S., Nam K.H., Lee D., Lee S.S., Ko S.H. (2012). Highly Stretchable and Highly Conductive Metal Electrode by Very Long Metal Nanowire Percolation Network. Adv. Mater..

[25] Chen J., Zheng J., Gao Q., Zhang J., Zhang J., Omisore O.M., Wang L., Li H. (2018). Polydimethylsiloxane (PDMS)-Based Flexible Resistive Strain Sensors for Wearable Applications. Appl. Sci..

[26] Hammock M.L., Chortos A., Tee B.C.-K., Tok J.B.-H., Bao Z. (2013). 25th Anniversary Article: The Evolution of Electronic Skin (E-Skin): A Brief History, Design Considerations, and Recent Progress. Adv. Mater..

[27] Sun Y., Rogers J.A. (2007). Structural forms of single crystal semiconductor nanoribbons for high-performance stretchable electronics. J. Mater. Chem..

[28] Zhang A., Shyam A.B., Cunningham A.M., Williams C., Brissenden A., Bartley A., Amsden B., Docoslis A., Kontopoulou M., Ameri S.K. (2023). Adhesive Wearable Sensors for Electroencephalography from Hairy Scalp. Adv. Healthc. Mater..

[29] Yang J., Liu L., Zhang D., Zhang H., Ma J., Zheng J., Wang C. (2023). Dual-Stage Surficial Microstructure to Enhance the Sensitivity of MXene Pressure Sensors for Human Physiological Signal Acquisition. ACS Appl. Mater. Interfaces.

[30] Zhao X., Mei D., Tang G., Zhao C., Wang J., Luo M., Li L., Wang Y. (2023). Strain and Pressure Sensors Based on MWCNT/PDMS for Human Motion/Perception Detection. Polymers.

[31] Hua Y., Guan M., Xia L., Chen Y., Mai J., Zhao C., Liao C. (2023). Highly Stretchable and Robust Electrochemical Sensor Based on 3D Graphene Oxide–CNT Composite for Detecting Ammonium in Sweat. Biosensors.

[32] Han S.-T., Peng H., Sun Q., Venkatesh S., Chung K.-S., Lau S.C., Zhou Y., Roy V.A.L. (2017). An Overview of the Development of Flexible Sensors. Adv. Mater..

[33] Zazoum B., Batoo K.M., Khan M.A.A. (2022). Recent Advances in Flexible Sensors and Their Applications. Sensors.

[34] Rim Y.S., Bae S.H., Chen H., De Marco N., Yang Y. (2016). Recent Progress in Materials and Devices toward Printable and Flexible Sensors. Adv. Mater..

[35] Zhang T., Chai Y., Wang S., Yu J., Jiang S., Zhu W., Fang Z., Li B. (2023). Recent Study Advances in Flexible Sensors Based on Polyimides. Sensors.

[36] Yi C., Li W., Shi S., He K., Ma P., Chen M., Yang C. (2020). High-temperature-resistant and colorless polyimide: Preparations, properties, and applications. Sol. Energy.

[37] Wu W.-Y., Hsu Y.-H., Chen Y.-F., Wu Y.-R., Liu H.-W., Tu T.-Y., Chao P.P.-C., Tan C.-S., Horng R.-H. (2021). Wearable Devices Made of a Wireless Vertical-Type Light-Emitting Diode Package on a Flexible Polyimide Substrate with a Conductive Layer. ACS Appl. Electron. Mater..

[38] Lee D.U., Kim S.-C., Choi D.Y., Jung W.-K., Moon M.J. (2023). Basic amino acid-mediated cationic amphiphilic surfaces for antimicrobial pH monitoring sensor with wound healing effects. Biomater. Res..

[39] Kou L., Ye N., Waheed A., Auliya R.Z., Wu C., Ooi P.C., Li F. (2023). High sensitivity and wide response range artificial synapse based on polyimide with embedded graphene quantum dots. Sci. Rep..

[40] Liu W., Huang Y., Peng Y., Walczak M.S., Wang D., Chen Q., Liu Z., Li L. (2020). Stable Wearable Strain Sensors on Textiles by Direct Laser Writing of Graphene. ACS Appl. Nano Mater..

[41] Lin J., Peng Z., Liu Y., Ruiz-Zepeda F., Ye R., Samuel E.L.G., Yacaman M.J., Yakobson B.I., Tour J.M. (2014). Laser-induced porous graphene films from commercial polymers. Nat. Commun..

[42] Alemán J.V., Chadwick A.V., He J., Hess M., Horie K., Jones R.G., Kratochvíl P., Meisel I., Mita I., Moad G. (2007). Definitions of terms relating to the structure and processing of sols, gels, networks, and inorganic-organic hybrid materials (IUPAC Recommendations 2007). Pure Appl. Chem..

[43] Abrisham M., Panahi-Sarmad M., Sadeghi G.M.M., Arjmand M., Dehghan P., Amirkiai A. (2020). Microstructural design for enhanced mechanical property and shape memory behavior of polyurethane nanocomposites: Role of carbon nanotube, montmorillonite, and their hybrid fillers. Polym. Test..

[44] Amirkiai A., Panahi-Sarmad M., Sadeghi G.M.M., Arjmand M., Abrisham M., Dehghan P., Nazockdast H. (2020). Microstructural design for enhanced mechanical and shape memory performance of polyurethane nanocomposites: Role of hybrid nanofillers of montmorillonite and halloysite nanotube. Appl. Clay Sci..

[45] Zhang Y., Zhao Y., Zhai W., Zheng G., Ji Y., Dai K., Mi L., Zhang D., Liu C., Shen C. (2021). Multifunctional interlocked e-skin based on elastic micropattern array facilely prepared by hot-air-gun. Chem. Eng. J..

[46] Tran M.T., Tung T.T., Sachan A., Losic D., Castro M., Feller J.F. (2020). 3D Sprayed Polyurethane Functionalized Graphene / Carbon Nanotubes Hybrid Architectures to Enhance the Piezo-Resistive Response of Quantum Resistive Pressure Sensors. Carbon.

[47] Zhang S., Sun K., Liu H., Chen X., Zheng Y., Shi X., Zhang D., Mi L., Liu C., Shen C. (2020). Enhanced piezoresistive performance of conductive WPU/CNT composite foam through incorporating brittle cellulose nanocrystal. Chem. Eng. J..

[48] Zhao Y., Ren M., Shang Y., Li J., Wang S., Zhai W., Zheng G., Dai K., Liu C., Shen C. (2020). Ultra-sensitive and durable strain sensor with sandwich structure and excellent anti-interference ability for wearable electronic skins. Compos. Sci. Technol..

[49] Chen T., Xie Y., Wang Z., Lou J., Liu D., Xu R., Cui Z., Li S., Panahi-Sarmad M., Xiao X. (2021). Recent Advances of Flexible Strain Sensors Based on Conductive Fillers and Thermoplastic Polyurethane Matrixes. ACS Appl. Polym. Mater..

[50] Wu S.-D., Hsu S., Ketelsen B., Bittinger S.C., Schlicke H., Weller H., Vossmeyer T. (2023). Fabrication of Eco-Friendly Wearable Strain Sensor Arrays via Facile Contact Printing for Healthcare Applications (Small Methods 9/2023). Small Methods.

[51] Bai L., Jin Y., Shang X., Jin H., Zhou Y., Shi L. (2021). Highly synergistic, electromechanical and mechanochromic dual-sensing ionic skin with multiple monitoring, antibacterial, self-healing, and anti-freezing functions. J. Mater. Chem. A.

[52] Hu X., Wang J., Song S., Gan W., Li W., Qi H., Zhang Y. (2024). Ionic conductive konjac glucomannan/liquid crystal cellulose composite hydrogels with dual sensing of photo- and electro-signals capacities as wearable strain sensors. Int. J. Biol. Macromol..

[53] Zhang P., Tong X., Gao Y., Qian Z., Ren R., Bian C., Wang J., Cai G. (2023). A Sensing and Stretchable Polymer-Dispersed Liquid Crystal Device Based on Spiderweb-Inspired Silver Nanowires-Micromesh Transparent Electrode. Adv. Funct. Mater..

[54] Tanaka T. (1978). Collapse of Gels and the Critical Endpoint. Phys. Rev. Lett..

[55] Detamornrat U., Parrilla M., Domínguez-Robles J., Anjani Q.K., Larrañeta E., De Wael K., Donnelly R.F. (2023). Transdermal on-demand drug delivery based on an iontophoretic hollow microneedle array system. Lab Chip.

[56] Yin R., Zhang C., Shao J., Chen Y., Yin A., Feng Q., Chen S., Peng F., Ma X., Xu C.-Y. (2023). Integration of flexible, recyclable, and transient gelatin hydrogels toward multifunctional electronics. J. Mater. Sci. Technol..

[57] Yang Z., Bao G., Huo R., Jiang S., Yang X., Ni X., Mongeau L., Long R., Li J. (2023). Programming hydrogel adhesion with engineered polymer network topology. Proc. Natl. Acad. Sci. USA.

[58] Xu L., Liu S., Zhu L., Liu Y., Li N., Shi X., Jiao T., Qin Z. (2023). Hydroxypropyl methyl cellulose reinforced conducting polymer hydrogels with ultra-stretchability and low hysteresis as highly sensitive strain sensors for wearable health monitoring. Int. J. Biol. Macromol..

[59] Peng Y., Peng H., Chen Z., Zhang J. (2024). Ultrasensitive Soft Sensor from Anisotropic Conductive Biphasic Liquid Metal-Polymer Gels. Adv. Mater..

[60] Li Y., Lu D., Wong C.P. (2010). Intrinsically Conducting Polymers (ICPs). Electrical Conductive Adhesives with Nanotechnologies.

[61] Farrell T.P., Kaner R.B. (2021). Conducting Polymers. Encyclopedia of Polymeric Nanomaterials.

[62] Ouyang J. (2021). Application of intrinsically conducting polymers in flexible electronics. SmartMat.

[63] Li T., Liang B., Ye Z., Zhang L., Xu S., Tu T., Zhang Y., Cai Y., Zhang B., Fang L. (2022). An integrated and conductive hydrogel-paper patch for simultaneous sensing of Chemical–Electrophysiological signals. Biosens. Bioelectron..

[64] Picchio M.L., Gallastegui A., Casado N., Lopez-Larrea N., Marchiori B., del Agua I., Criado-Gonzalez M., Mantione D., Minari R.J., Mecerreyes D. (2022). Mixed Ionic and Electronic Conducting Eutectogels for 3D-Printable Wearable Sensors and Bioelectrodes. Adv. Mater. Technol..

[65] Sha L., Chen Z., Chen Z., Zhang A., Yang Z. (2016). Polylactic Acid Based Nanocomposites: Promising Safe and Biodegradable Materials in Biomedical Field. Int. J. Polym. Sci..

[66] Zarei M., Lee G., Lee S.G., Cho K. (2023). Advances in Biodegradable Electronic Skin: Material Progress and Recent Applications in Sensing, Robotics, and Human–Machine Interfaces. Adv. Mater..

[67] Sreejith S., Joseph L.L., Kollem S., Vijumon V.T., Ajayan J. (2023). Biodegradable sensors: A comprehensive review. Measurement.

[68] Zhu J., Wen H., Zhang H., Huang P., Liu L., Hu H. (2023). Recent advances in biodegradable electronics- from fundament to the next-generation multi-functional, medical and environmental device. Sustain. Mater. Technol..

[69] Yin L., Farimani A.B., Min K., Vishal N., Lam J., Lee Y.K., Aluru N.R., Rogers J.A. (2015). Mechanisms for Hydrolysis of Silicon Nanomembranes as Used in Bioresorbable Electronics. Adv. Mater..

[70] Hosseini E.S., Dervin S., Ganguly P., Dahiya R. (2020). Biodegradable Materials for Sustainable Health Monitoring Devices. ACS Appl. Bio Mater..

[71] Guo Y., Zhong M., Fang Z., Wan P., Yu G. (2019). A Wearable Transient Pressure Sensor Made with MXene Nanosheets for Sensitive Broad-Range Human–Machine Interfacing. Nano Lett..

[72] Selamneni V., Sahatiya P. (2020). Bolometric Effect Enhanced Ultrafast Graphene Based Do-It-Yourself Wearable Respiration Sensor for Personal Healthcare Monitoring. IEEE Sens. J..

[73] Chao M., He L., Gong M., Li N., Li X., Peng L., Shi F., Zhang L., Wan P. (2021). Breathable Ti_3_C_2_T_x_ MXene/Protein Nanocomposites for Ultrasensitive Medical Pressure Sensor with Degradability in Solvents. ACS Nano.

[74] Xu M., Yadavalli V.K. (2019). Flexible Biosensors for the Impedimetric Detection of Protein Targets Using Silk-Conductive Polymer Biocomposites. ACS Sens..

[75] Jadoun S. (2024). Synthesis, Mechanism, and Applications of Self-healing Materials. Biomed. Mater. Devices.

[76] Wool R.P. (2008). Self-healing materials: A review. Soft Matter.

[77] Arani A.G., Miralaei N., Farazin A., Mohammadimehr M. (2023). An extensive review of the repair behavior of smart self-healing polymer matrix composites. J. Mater. Res..

[78] Thangavel G., Tan M.W.M., Lee P.S. (2019). Advances in self-healing supramolecular soft materials and nanocomposites. Nano Converg..

[79] Wan Y., Li X.-C., Yuan H., Liu D., Lai W.-Y. (2024). Self-Healing Elastic Electronics: Materials Design, Mechanisms, and Applications. Adv. Funct. Mater..

[80] Yimyai T., Crespy D., Pena-Francesch A. (2023). Self-Healing Photochromic Elastomer Composites for Wearable UV-Sensors. Adv. Funct. Mater..

[81] Omidian H., Chowdhury S.D. (2023). High-Performing Conductive Hydrogels for Wearable Applications. Gels.

[82] Luo Y., Li J., Ding Q., Wang H., Liu C., Wu J. (2023). Functionalized Hydrogel-Based Wearable Gas and Humidity Sensors. Nano-Micro Lett..

[83] Chen S., Fan S., Chan H., Qiao Z., Qi J., Wu Z., Yeo J.C., Lim C.T. (2023). Liquid Metal Functionalization Innovations in Wearables and Soft Robotics for Smart Healthcare Applications. Adv. Funct. Mater..

[84] Yang J., Cheng J., Qi G., Wang B. (2023). Ultrastretchable, Multihealable, and Highly Sensitive Strain Sensor Based on a Double Cross-Linked MXene Hydrogel. ACS Appl. Mater. Interfaces.

[85] Seong M., Kondaveeti S., Choi G., Kim S., Kim J., Kang M., Jeong H.E. (2023). 3D Printable Self-Adhesive and Self-Healing Ionotronic Hydrogels for Wearable Healthcare Devices. ACS Appl. Mater. Interfaces.

[86] Zhou X., Kateb P., Fan J., Kim J., Lodygensky G.A., Amilhon B., Pasini D., Cicoira F. (2023). Conducting Polymer Films and Bioelectrodes Combining High Adhesion and Electro-Mechanical Self-Healing. J. Mater. Chem. C.

[87] Sun S., Yuan R., Ling S., Zhou T., Wu Z., Fu M., He H., Li X., Zhang C. (2024). Self-Healable, Self-Adhesive and Degradable MXene-Based Multifunctional Hydrogel for Flexible Epidermal Sensors. ACS Appl. Mater. Interfaces.

[88] Wang Q., Yu Y., Liu J. (2018). Preparations, Characteristics and Applications of the Functional Liquid Metal Materials. Adv. Eng. Mater..

[89] Won P., Jeong S., Majidi C., Ko S.H. (2021). Recent advances in liquid-metal-based wearable electronics and materials. iScience.

[90] Ma J., Krisnadi F., Vong M.H., Kong M., Awartani O.M., Dickey M.D. (2023). Shaping a Soft Future: Patterning Liquid Metals. Adv. Mater..

[91] Kim S., Saito M., Wei Y., Bhuyan P., Choe M., Fujie T., Mondal K., Park S. (2023). Stretchable and wearable polymeric heaters and strain sensors fabricated using liquid metals. Sens. Actuators A Phys..

[92] Kim H., Zan G., Seo Y., Lee S., Park C. (2023). Stimuli-Responsive Liquid Metal Hybrids for Human-Interactive Electronics. Adv. Funct. Mater..

[93] Wang L., Lai R., Zhang L., Zeng M., Fu L. (2022). Emerging Liquid Metal Biomaterials: From Design to Application. Adv. Mater..

[94] Yu L., Yeo J.C., Soon R.H., Yeo T., Lee H.H., Lim C.T. (2018). Highly Stretchable, Weavable, and Washable Piezoresistive Microfiber Sensors. ACS Appl. Mater. Interfaces.

[95] Liang S., Li Y., Chen Y., Yang J., Zhu T., Zhu D., He C., Liu Y., Handschuh-Wang S., Zhou X. (2017). Liquid metal sponges for mechanically durable, all-soft, electrical conductors. J. Mater. Chem. C.

[96] Liu H., Xin Y., Lou Y., Peng Y., Wei L., Zhang J. (2020). Liquid metal gradient fibers with reversible thermal programmability. Mater. Horiz..

[97] Deng X., Chen G., Liao Y., Lu X., Hu S., Gan T., Handschuh-Wang S., Zhang X. (2022). Self-Healable and Recyclable Dual-Shape Memory Liquid Metal–Elastomer Composites. Polymers.

[98] Liu R., Gong L., Zhu X., Zhu S., Wu X., Xue T., Yan L., Du J., Gu Z. (2022). Transformable Gallium-Based Liquid Metal Nanoparticles for Tumor Radiotherapy Sensitization. Adv. Healthc. Mater..

[99] Yi P., Zou H., Yu Y., Li X., Li Z., Deng G., Chen C., Fang M., He J., Sun X. (2022). MXene-Reinforced Liquid Metal/Polymer Fibers via Interface Engineering for Wearable Multifunctional Textiles. ACS Nano.

[100] Cao L., Yu D., Xia Z., Wan H., Liu C., Yin T., He Z. (2020). Ferromagnetic Liquid Metal Putty-Like Material with Transformed Shape and Reconfigurable Polarity. Adv. Mater..

[101] Markvicka E.J., Bartlett M.D., Huang X., Majidi C. (2018). An autonomously electrically self-healing liquid metal–elastomer composite for robust soft-matter robotics and electronics. Nat. Mater..

[102] Zhu P., Luo X., Lin X., Qiu Z., Chen R., Wang X., Wang Y., Deng Y., Mao Y. (2023). A self-healable, recyclable, and flexible thermoelectric device for wearable energy harvesting and personal thermal management. Energy Convers. Manag..

[103] Feng X., Wang C., Shang S., Liu H., Huang X., Jiang J., Song Z., Zhang H. (2023). Self-healing, EMI shielding, and antibacterial properties of recyclable cellulose liquid metal hydrogel sensor. Carbohydr. Polym..

[104] Min S., Kim D.H., Joe D.J., Kim B.W., Jung Y.H., Lee J.H., Lee B., Doh I., An J., Youn Y. (2023). Clinical Validation of a Wearable Piezoelectric Blood-Pressure Sensor for Continuous Health Monitoring. Adv. Mater..

[105] Sun J., Xiu K., Wang Z., Hu N., Zhao L., Zhu H., Kong F., Xiao J., Cheng L., Bi X. (2023). Multifunctional wearable humidity and pressure sensors based on biocompatible graphene/bacterial cellulose bioaerogel for wireless monitoring and early warning of sleep apnea syndrome. Nano Energy.

[106] Zhao H., Zhang L., Deng T., Li C. (2023). High-performance sensing, breathable, and biodegradable integrated wearable sweat biosensors for a wireless glucose early warning system. J. Mater. Chem. A.

[107] Engel E., Hill A.C., Granchelli A., Lockhart G.C., Lyng G., Kaye L., Lyson H., Bowler R.P. (2023). Wearable Sensors for Early Detection of Copd Exacerbations. Chest.

[108] Quinn H., Davis J. (2023). Detecting the early onset of hyponatremia: An opportunity for wearable sensors?. Curr. Opin. Electrochem..

[109] Khijmatgar S., Yong J., Rübsamen N., Lorusso F., Rai P., Cenzato N., Gaffuri F., Del Fabbro M., Tartaglia G.M. (2024). Salivary biomarkers for early detection of oral squamous cell carcinoma (OSCC) and head/neck squamous cell carcinoma (HNSCC): A systematic review and network meta-analysis. Jpn. Dent. Sci. Rev..

[110] Linh V.T.N., Kim H., Lee M.-Y., Mun J., Kim Y., Jeong B.-H., Park S.-G., Kim D.-H., Rho J., Jung H.S. (2024). 3D plasmonic hexaplex paper sensor for label-free human saliva sensing and machine learning-assisted early-stage lung cancer screening. Biosens. Bioelectron..

[111] Ates H.C., Nguyen P.Q., Gonzalez-Macia L., Morales-Narváez E., Güder F., Collins J.J., Dincer C. (2022). End-to-end design of wearable sensors. Nat. Rev. Mater..

[112] Abramson A., Chan C.T., Khan Y., Mermin-Bunnell A., Matsuhisa N., Fong R., Shad R., Hiesinger W., Mallick P., Gambhir S.S. (2022). A flexible electronic strain sensor for the real-time monitoring of tumor regression. Sci. Adv..

[113] Chun K.-Y., Seo S., Han C.-S. (2022). A Wearable All-Gel Multimodal Cutaneous Sensor Enabling Simultaneous Single-Site Monitoring of Cardiac-Related Biophysical Signals. Adv. Mater..

[114] Bi Y., Sun M., Wang J., Zhu Z., Bai J., Emran M.Y., Kotb A., Bo X., Zhou M. (2023). Universal Fully Integrated Wearable Sensor Arrays for the Multiple Electrolyte and Metabolite Monitoring in Raw Sweat, Saliva, or Urine. Anal. Chem..

[115] Quer G., Radin J.M., Gadaleta M., Baca-Motes K., Ariniello L., Ramos E., Kheterpal V., Topol E.J., Steinhubl S.R. (2021). Wearable sensor data and self-reported symptoms for COVID-19 detection. Nat. Med..

[116] Erdem A., Eksin E., Senturk H., Yildiz E., Maral M. (2024). Recent developments in wearable biosensors for healthcare and biomedical applications. TrAC Trends Anal. Chem..

[117] Sharma A., Badea M., Tiwari S., Marty J.L. (2021). Wearable Biosensors: An Alternative and Practical Approach in Healthcare and Disease Monitoring. Molecules.

[118] Zahradka N., Geoghan S., Watson H., Goldberg E., Wolfberg A., Wilkes M. (2023). Assessment of Remote Vital Sign Monitoring and Alarms in a Real-World Healthcare at Home Dataset. Bioengineering.

[119] Hussain T., Ullah S., Fernández-García R., Gil I. (2023). Wearable Sensors for Respiration Monitoring: A Review. Sensors.

[120] Huang X., Zheng Z., Wang H., Xu W., Wu M., Wang M., Chen C., Wan L., Du R., Zhu T. (2024). A Freeze-Resistant, Highly Stretchable and Biocompatible Organohydrogel for Non-Delayed Wearable Sensing at Ultralow-Temperatures. Adv. Funct. Mater..

[121] Dang X., Fu Y., Wang X. (2024). A temperature and pressure dual-responsive, stretchable, healable, adhesive, and biocompatible carboxymethyl cellulose-based conductive hydrogels for flexible wearable strain sensor. Biosens. Bioelectron..

[122] Hong Q., Liu T., Guo X., Yan Z., Li W., Liu L., Wang D., Hong W., Qian Z., Zhang A. (2024). 3D dual-mode tactile sensor with decoupled temperature and pressure sensing: Toward biological skins for wearable devices and smart robotics. Sens. Actuators B Chem..

[123] Wang J., Sun Y., Jia P., Su J., Zhang X., Wu N., Yu H., Song Y., Zhou J. (2023). Wearable nanocomposite hydrogel temperature sensor based on thermally-switchable and mechanical-deformation-insensitive structural colors. Chem. Eng. J..

[124] Faridi S., Allen R.W., Brook R.D., Yousefian F., Hassanvand M.S., Carlsten C. (2023). An updated systematic review and meta-analysis on portable air cleaners and blood pressure: Recommendations for users and manufacturers. Ecotoxicol. Environ. Saf..

[125] Ismail S.N.A., Nayan N.A., Haniff M.A.S.M., Jaafar R., May Z. (2023). Wearable Two-Dimensional Nanomaterial-Based Flexible Sensors for Blood Pressure Monitoring: A Review. Nanomaterials.

[126] Zhang Q., Zhang H., Liang J., Zhao X., Li B., Zang J., Gao L., Zhang Z.D., Xue C. (2023). Ti_3_C_2_T*_x_*-MXene/PET textile-based flexible pressure sensor for wearable pulse monitoring. J. Mater. Chem. C.

[127] Shi C., Zhang H., Ni X., Wang K. (2023). An FBG-Based Sensor with Both Wearable and Handheld Forms for Carotid Arterial Pulse Waveform Measurement. IEEE Trans. Instrum. Meas..

[128] Heimark S., Hove C., Stepanov A., Boysen E.S., Gløersen Ø., Bøtke-Rasmussen K.G., Gravdal H.J., Narayanapillai K., Larstorp A.C.K., Seeberg T.M. (2023). 24-Hour Ambulatory Blood Pressure Measurement Using a Pulse Arrival Time Based Blood Pressure Model by a Prototype Wearable Sensor. J. Hypertens..

[129] Kumar R., Fu J., Ortiz B.L., Cao X., Shedden K., Choi S.W. (2024). Dyadic and Individual Variation in 24-Hour Heart Rates of Cancer Patients and Their Caregivers. Bioengineering.

[130] Marasco I., Niro G., Demir S.M., Marzano L., Fachechi L., Rizzi F., Demarchi D., Ros P.M., D’orazio A., Grande M. (2023). Wearable Heart Rate Monitoring Device Communicating in 5G ISM Band for IoHT. Bioengineering.

[131] Yang Y., Xu S., Gai Y., Zhang B., Chen L. (2022). Recent Progresses in Lanthanide Metal-Organic Frameworks (Ln-MOFs) as Chemical Sensors for Ions, Antibiotics and Amino Acids. Chin. J. Struct. Chem..

[132] Kaiqiang W., Xingyang L. (2020). Wearable pressure sensor for athletes’ full-range motion signal monitoring. Mater. Res. Express.

[133] Warmerdam E., Orth M., Pohlemann T., Ganse B. (2023). Gait Analysis to Monitor Fracture Healing of the Lower Leg. Bioengineering.

[134] Lee C., Ahn J., Lee B.-C. (2023). A Systematic Review of the Long-Term Effects of Using Smartphone- and Tablet-Based Rehabilitation Technology for Balance and Gait Training and Exercise Programs. Bioengineering.

[135] May D.S., Tueth L.E., Earhart G.M., Mazzoni P. (2023). Using Wearable Sensors to Assess Freezing of Gait in the Real World. Bioengineering.

[136] Pieruccini-Faria F., Black S.E., Masellis M., Smith E.E., Almeida Q.J., Li K.Z.H., Bherer L., Camicioli R., Montero-Odasso M. (2021). Gait variability across neurodegenerative and cognitive disorders: Results from the Canadian Consortium of Neurodegeneration in Aging (CCNA) and the Gait and Brain Study. Alzheimer’s Dement..

[137] Cicirelli G., Impedovo D., Dentamaro V., Marani R., Pirlo G., D’Orazio T.R. (2021). Human Gait Analysis in Neurodegenerative Diseases: A Review. IEEE J. Biomed. Health Inform..

[138] Marković V., Stanković I., Radovanović S., Petrović I., Lukić M.J., Mišković N.D., Svetel M., Kostić V. (2022). Gait alterations in Parkinson’s disease at the stage of hemiparkinsonism—A longitudinal study. PLoS ONE.

[139] Ahn J.W., Ku Y., Kim H.C. (2019). A Novel Wearable EEG and ECG Recording System for Stress Assessment. Sensors.

[140] Pourmohammadi S., Maleki A. (2020). Stress detection using ECG and EMG signals: A comprehensive study. Comput. Methods Programs Biomed..

[141] Umer W. (2022). Simultaneous monitoring of physical and mental stress for construction tasks using physiological measures. J. Build. Eng..

[142] Ritsert F., Elgendi M., Galli V., Menon C. (2022). Heart and Breathing Rate Variations as Biomarkers for Anxiety Detection. Bioengineering.

[143] Zhu G., Huang Y., Wang X., Wang X., Li F., Pan S. (2022). Basic Theory of EEG. Multi-Modal EEG Monitoring of Severely Neurologically Ill Patients.

[144] Li F., Wang X., Li F., Pan S. (2022). Application of Multimodal EEG in the Determination of Brain Death. Multi-Modal EEG Monitoring of Severely Neurologically Ill Patients.

[145] Wang Y., Lin Y., Wang X., Li F., Pan S. (2022). Patterns and Clinical Significance of Abnormal Sleep EEG. Multi-Modal EEG Monitoring of Severely Neurologically Ill Patients.

[146] Zhou Y., Li F., Wang X., Li F., Pan S. (2022). Application of aEEG in Severely Ill Patients. Multi-Modal EEG Monitoring of Severely Neurologically Ill Patients.

[147] Wang X., Yan Y., Wang X., Li F., Pan S. (2022). Abnormal EEG Background Activity. Multi-Modal EEG Monitoring of Severely Neurologically Ill Patients.

[148] Li F., Huang Z., Wang X., Li F., Pan S. (2022). qEEG Monitoring System in Severely Ill Patients. Multi-Modal EEG Monitoring of Severely Neurologically Ill Patients.

[149] Wang X., Li J., Jing W., Wang X., Li F., Pan S. (2022). Application of Multimodal EEG in SE. Multi-Modal EEG Monitoring of Severely Neurologically Ill Patients.

[150] Zhuang Y., Ge Q., Li Q., Xu L., Geng X., Wang R., He J. (2024). Combined behavioral and EEG evidence for the 70 Hz frequency selection of short-term spinal cord stimulation in disorders of consciousness. CNS Neurosci. Ther..

[151] Mehmood I., Li H., Qarout Y., Umer W., Anwer S., Wu H., Hussain M., Antwi-Afari M.F. (2023). Deep learning-based construction equipment operators’ mental fatigue classification using wearable EEG sensor data. Adv. Eng. Inform..

[152] Zhao S., Dai G., Li J., Zhu X., Huang X., Li Y., Tan M., Wang L., Fang P., Chen X. (2024). An interpretable model based on graph learning for diagnosis of Parkinson’s disease with voice-related EEG. npj Digit. Med..

[153] Zhang Z., Meng Q., Jin L., Wang H., Hou H. (2023). A Novel EEG-Based Graph Convolution Network for Depression Detection: Incorporating Secondary Subject Partitioning and Attention Mechanism. Expert Syst. Appl..

[154] Srinivasan S., Johnson S.D. (2024). A novel approach to schizophrenia Detection: Optimized preprocessing and deep learning analysis of multichannel EEG data. Expert Syst. Appl..

[155] Chen J., Cui Y., Wang H., He E., Alhudhaif A. (2024). Deep learning approach for detection of unfavorable driving state based on multiple phase synchronization between multi-channel EEG signals. Inf. Sci..

[156] Song Y., Fan C., Mao X. (2024). Optimization of Epilepsy Detection Method Based on Dynamic EEG Channel Screening. Neural Netw..

[157] Li A., Deng Z., Zhang W., Xiao Z., Choi K.-S., Liu Y., Hu S., Wang S. (2024). Multiview Transfer Representation Learning with TSK Fuzzy System for EEG Epilepsy Detection. IEEE Trans. Fuzzy Syst..

[158] Anita M., MeenaKowshalya A.M. (2024). Automatic epileptic seizure detection using MSA-DCNN and LSTM techniques with EEG signals. Expert Syst. Appl..

[159] Han Y., Zeng X., Hua L., Quan X., Chen Y., Zhou M., Chuang Y., Li Y., Wang S., Shen X. (2024). The fusion of multi-omics profile and multimodal EEG data contributes to the personalized diagnostic strategy for neurocognitive disorders. Microbiome.

[160] Deng J., Sun B., Kavcic V., Liu M., Giordani B., Li T. (2024). Novel methodology for detection and prediction of mild cognitive impairment using resting-state EEG. Alzheimer’s Dement..

[161] Ntolkeras G., Makaram N., Bernabei M., De La Vega A.C., Bolton J., Madsen J.R., Stone S.S.D., Pearl P.L., Papadelis C., Grant E.P. (2024). Interictal EEG source connectivity to localize the epileptogenic zone in patients with drug-resistant epilepsy: A machine learning approach. Epilepsia.

[162] Bernabei J.M., Li A., Revell A.Y., Smith R.J., Gunnarsdottir K.M., Ong I.Z., Davis K.A., Sinha N., Sarma S., Litt B. (2023). Quantitative approaches to guide epilepsy surgery from intracranial EEG. Brain.

[163] Kučikienė D., Rajkumar R., Timpte K., Heckelmann J., Neuner I., Weber Y., Wolking S. (2024). EEG microstates show different features in focal epilepsy and psychogenic nonepileptic seizures. Epilepsia.

[164] Chai X., Cao T., He Q., Wang N., Zhang X., Shan X., Lv Z., Tu W., Yang Y., Zhao J. (2024). Brain–computer interface digital prescription for neurological disorders. CNS Neurosci. Ther..

[165] Friedman P.A. (2024). The Electrocardiogram at 100 Years: History and Future. Circulation.

[166] Pham H.N., Holmstrom L., Chugh H., Uy-Evanado A., Nakamura K., Zhang Z., Salvucci A., Jui J., Reinier K., Chugh S.S. (2024). Dynamic electrocardiogram changes are a novel risk marker for sudden cardiac death. Eur. Heart J..

[167] Soh C.H., de Sá A.G.C., Potter E., Halabi A., Ascher D.B., Marwick T.H. (2024). Use of the energy waveform electrocardiogram to detect subclinical left ventricular dysfunction in patients with type 2 diabetes mellitus. Cardiovasc. Diabetol..

[168] Zhang S., Lian C., Xu B., Su Y., Alhudhaif A. (2024). 12-Lead ECG Signal Classification for Detecting ECG Arrhythmia via An Information Bottleneck-Based Multi-Scale Network. Inf. Sci..

[169] Lee E., Ito S., Miranda W.R., Lopez-Jimenez F., Kane G.C., Asirvatham S.J., Noseworthy P.A., Friedman P.A., Carter R.E., Borlaug B.A. (2024). Artificial intelligence-enabled ECG for left ventricular diastolic function and filling pressure. npj Digit. Med..

[170] Chen J., Huang S., Zhang Y., Chang Q., Zhang Y., Li D., Qiu J., Hu L., Peng X., Du Y. (2024). Congenital heart disease detection by pediatric electrocardiogram based deep learning integrated with human concepts. Nat. Commun..

[171] Xiong P., Wu C., Zhou H., Song A., Hu L., Liu X.P. (2018). Design of an accurate end-of-arm force display system based on wearable arm gesture sensors and EMG sensors. Inf. Fusion.

[172] Chand S., McDaid A., Lu Y. (2023). Dynamic muscle fatigue assessment using s-EMG technology towards human-centric human-robot collaboration. J. Manuf. Syst..

[173] Jonkman A.H., Warnaar R.S.P., Baccinelli W., Carbon N.M., D’cruz R.F., Doorduin J., van Doorn J.L.M., Elshof J., Estrada-Petrocelli L., Graßhoff J. (2024). Analysis and applications of respiratory surface EMG: Report of a round table meeting. Crit. Care.

[174] Fan J., Jiang X., Liu X., Meng L., Jia F., Dai C. (2024). Surface EMG feature disentanglement for robust pattern recognition. Expert Syst. Appl..

[175] Park J., Jeong J., Kang M., Pritish N., Cho Y., Ha J., Yea J., Jang K.-I., Kim H., Hwang J. (2023). Imperceptive and reusable dermal surface EMG for lower extremity neuro-prosthetic control and clinical assessment. npj Flex. Electron..

[176] Li J.-W., Chen H.-F., Liu Y.-Z., Wang J.-H., Lu M.-C., Chiu C.-W. (2024). Photocurable 3D-printed AgNPs/Graphene/Polymer nanocomposites with high flexibility and stretchability for ECG and EMG smart clothing. Chem. Eng. J..

[177] (2021). Inertial Sensors and Inertial Measurement Units. Pedestrian Inertial Navigation with Self-Contained Aiding.

[178] Chatterjee G., Latorre L., Mailly F., Nouet P., Hachelef N., Oudea C. (2017). Smart-MEMS based inertial measurement units: Gyro-free approach to improve the grade. Microsyst. Technol..

[179] Washabaugh E.P., Kalyanaraman T., Adamczyk P.G., Claflin E.S., Krishnan C. (2017). Validity and repeatability of inertial measurement units for measuring gait parameters. Gait Posture.

[180] Young F., Mason R., Morris R.E., Stuart S., Godfrey A. (2023). IoT-Enabled Gait Assessment: The Next Step for Habitual Monitoring. Sensors.

[181] Rahmani M.H., Berkvens R., Weyn M. (2021). Chest-Worn Inertial Sensors: A Survey of Applications and Methods. Sensors.

[182] Figueira V., Silva S., Costa I., Campos B., Salgado J., Pinho L., Freitas M., Carvalho P., Marques J., Pinho F. (2024). Wearables for Monitoring and Postural Feedback in the Work Context: A Scoping Review. Sensors.

[183] Blair S., Duthie G., Robertson S., Hopkins W., Ball K. (2018). Concurrent validation of an inertial measurement system to quantify kicking biomechanics in four football codes. J. Biomech..

[184] Xia K., Chen X., Chang X., Liu C., Guo L., Xu X., Lv F., Wang Y., Sun H., Zhou J. (2022). Hand Exoskeleton Design and Human–Machine Interaction Strategies for Rehabilitation. Bioengineering.

[185] Dey S., Schilling A.F. (2023). A Function Approximator Model for Robust Online Foot Angle Trajectory Prediction Using a Single IMU Sensor: Implication for Controlling Active Prosthetic Feet. IEEE Trans. Ind. Inform..

[186] Peng R., Wang Z., Lu P. (2023). AeCoM: An Aerial Continuum Manipulator With IMU-Based Kinematic Modeling and Tendon-Slacking Prevention. IEEE Trans. Syst. Man Cybern. Syst..

[187] Zhang X., Tricomi E., Missiroli F., Lotti N., Masia L. (2023). Real-Time Assistive Control via IMU Locomotion Mode Detection in a Soft Exosuit: An Effective Approach to Enhance Walking Metabolic Efficiency. IEEE/ASME Trans. Mechatron..

[188] Hong S., Yoon J., Ham Y., Lee B., Kim H. (2023). Monitoring safety behaviors of scaffolding workers using Gramian angular field convolution neural network based on IMU sensing data. Autom. Constr..

[189] Xu Z., Huang B., Jia B., Mao G. (2024). Enhancing WiFi Fingerprinting Localization Through a Co-Teaching Approach Using Crowdsourced Sequential RSS and IMU Data. IEEE Internet Things J..

[190] Huang H., Yang J., Fang X., Jiang H., Xie L. (2023). VariFi: Variational Inference for Indoor Pedestrian Localization and Tracking Using IMU and WiFi RSS. IEEE Internet Things J..

[191] Lee S., Lim Y., Lim K. (2024). Multimodal sensor fusion models for real-time exercise repetition counting with IMU sensors and respiration data. Inf. Fusion.

[192] Tan T., Wang D., Shull P.B., Halilaj E. (2023). IMU and Smartphone Camera Fusion for Knee Adduction and Knee Flexion Moment Estimation During Walking. IEEE Trans. Ind. Inform..

[193] Gomaa W., Khamis M.A. (2023). A perspective on human activity recognition from inertial motion data. Neural Comput. Appl..

[194] Edwards N.A., Talarico M.K., Chaudhari A., Mansfield C.J., Oñate J. (2023). Use of accelerometers and inertial measurement units to quantify movement of tactical athletes: A systematic review. Appl. Ergon..

[195] Demeco A., Frizziero A., Nuresi C., Buccino G., Pisani F., Martini C., Foresti R., Costantino C. (2023). Gait Alteration in Individual with Limb Loss: The Role of Inertial Sensors. Sensors.

[196] Kang G.E., Stout A., Waldon K., Kang S., Killeen A.L., Crisologo P.A., Siah M., Jupiter D., Najafi B., Vaziri A. (2022). Digital Biomarkers of Gait and Balance in Diabetic Foot, Measurable by Wearable Inertial Measurement Units: A Mini Review. Sensors.

[197] Liang W., Wang F., Fan A., Zhao W., Yao W., Yang P. (2023). Extended Application of Inertial Measurement Units in Biomechanics: From Activity Recognition to Force Estimation. Sensors.

[198] Weizman Y., Tirosh O., Fuss F.K., Tan A.M., Rutz E. (2022). Recent State of Wearable IMU Sensors Use in People Living with Spasticity: A Systematic Review. Sensors.

[199] Yogesh V., Buurke J.H., Veltink P.H., Baten C.T.M. (2023). Integrated UWB/MIMU Sensor System for Position Estimation towards an Accurate Analysis of Human Movement: A Technical Review. Sensors.

[200] Hu Y., Chatzilakou E., Pan Z., Traverso G., Yetisen A.K. (2024). Microneedle Sensors for Point-of-Care Diagnostics. Adv. Sci..

[201] Zhang X., Yao B., Hu Q., Hong Y., Wallace A., Reynolds K., Ramsey C., Maeder A., Reed R., Tang Y. (2020). Detection of biomarkers in body fluids using bioprobes based on aggregation-induced emission fluorogens. Mater. Chem. Front..

[202] Kho K.W., Qing K.Z.M., Shen Z.X., Ahmad I.B., Lim S.S.C., Mhaisalkar S., White T.J., Watt F., Soo K.C., Olivo M. (2008). Polymer-based microfluidics with surface-enhanced Raman-spectroscopy-active periodic metal nanostructures for biofluid analysis. J. Biomed. Opt..

[203] Guo J.-W., Yang Z.-W., Liu X.-L., Zhang L.-W., Guo W.-B., Zhang J., Ding L.-H. (2023). 2D Co metal-organic framework nanosheet as an oxidase-like nanozyme for sensitive biomolecule monitoring. Rare Met..

[204] Mustafa Y.L., Keirouz A., Leese H.S. (2022). Molecularly imprinted polymers in diagnostics: Accessing analytes in biofluids. J. Mater. Chem. B.

[205] Shende P., Trivedi R. (2021). Biofluidic material-based carriers: Potential systems for crossing cellular barriers. J. Control. Release.

[206] Li W., Wei H., Li N., Li S., Liu Y., Liu R., Zou W., Hu P., Zhang Z., Wang C. (2023). Rapid identification and quantification of diquat in biological fluids within 30 s using a portable Raman spectrometer. Biosens. Bioelectron..

[207] Li H., Gu S., Zhang Q., Song E., Kuang T., Chen F., Yu X., Chang L. (2021). Recent advances in biofluid detection with micro/nanostructured bioelectronic devices. Nanoscale.

[208] Chen Z.-B., Jin H.-H., Yang Z.-G., He D.-P. (2023). Recent advances on bioreceptors and metal nanomaterials-based electrochemical impedance spectroscopy biosensors. Rare Met..

[209] Jin C., Bai Z. (2022). MXene-Based Textile Sensors for Wearable Applications. ACS Sens..

[210] Saha T., Del Caño R., Mahato K., De la Paz E., Chen C., Ding S., Yin L., Wang J. (2023). Wearable Electrochemical Glucose Sensors in Diabetes Management: A Comprehensive Review. Chem. Rev..

[211] Saha T., Mukherjee S., Dickey M.D., Velev O.D. (2024). Harvesting and manipulating sweat and interstitial fluid in microfluidic devices. Lab Chip.

[212] Dervisevic M., Dervisevic E., Esser L., Easton C.D., Cadarso V.J., Voelcker N.H. (2023). Wearable microneedle array-based sensor for transdermal monitoring of pH levels in interstitial fluid. Biosens. Bioelectron..

[213] Samant P.P., Niedzwiecki M.M., Raviele N., Tran V., Mena-Lapaix J., Walker D.I., Felner E.I., Jones D.P., Miller G.W., Prausnitz M.R. (2020). Sampling interstitial fluid from human skin using a microneedle patch. Sci. Transl. Med..

[214] Min J., Tu J., Xu C., Lukas H., Shin S., Yang Y., Solomon S.A., Mukasa D., Gao W. (2023). Skin-Interfaced Wearable Sweat Sensors for Precision Medicine. Chem. Rev..

[215] Yang D.S., Ghaffari R., Rogers J.A. (2023). Sweat as a diagnostic biofluid. Science.

[216] Davis N., Heikenfeld J., Milla C., Javey A. (2024). The challenges and promise of sweat sensing. Nat. Biotechnol..

[217] Ha J.-H., Jeong Y., Ahn J., Hwang S.H., Jeon S., Kim D., Ko J., Kang B., Jung Y., Choi J. (2023). A wearable colorimetric sweat pH sensor-based smart textile for health state diagnosis. Mater. Horiz..

[218] Wang W., Chen Y., Xiao C., Xiao S., Wang C., Nie Q., Xu P., Chen J., You R., Zhang G. (2023). Flexible SERS wearable sensor based on nanocomposite hydrogel for detection of metabolites and pH in sweat. Chem. Eng. J..

[219] Xu Z., Qiao X., Tao R., Li Y., Zhao S., Cai Y., Luo X. (2023). A wearable sensor based on multifunctional conductive hydrogel for simultaneous accurate pH and tyrosine monitoring in sweat. Biosens. Bioelectron..

[220] Yoon J.H., Kim S.-M., Park H.J., Kim Y.K., Oh D.X., Cho H.-W., Lee K.G., Hwang S.Y., Park J., Choi B.G. (2020). Highly self-healable and flexible cable-type pH sensors for real-time monitoring of human fluids. Biosens. Bioelectron..

[221] Hajivand P., Jansen J.C., Pardo E., Armentano D., Mastropietro T.F., Azadmehr A. (2024). Application of metal-organic frameworks for sensing of VOCs and other volatile biomarkers. Coord. Chem. Rev..

[222] Pan D., Williams C.M., Decker J., Fletcher E., Sze S., Assadi S., Haigh R., Saleem B., Nazareth J., Garton N.J. (2023). Exhaled SARS-CoV-2 RNA viral load kinetics measured by facemask sampling associates with household transmission. Clin. Microbiol. Infect..

[223] Alsved M., Nyström K., Thuresson S., Nygren D., Patzi-Churqui M., Hussein T., Fraenkel C.-J., Medstrand P., Löndahl J. (2023). Infectivity of exhaled SARS-CoV-2 aerosols is sufficient to transmit COVID-19 within minutes. Sci. Rep..

[224] Hagan S., Tomlinson A. (2013). Tear Fluid Biomarker Profiling: A Review of Multiplex Bead Analysis. Ocul. Surf..

[225] Hamm-Alvarez S.F., Janga S.R., Edman M.C., Madrigal S., Shah M., Frousiakis S.E., Renduchintala K., Zhu J., Bricel S., Silka K. (2014). Tear Cathepsin S as a Candidate Biomarker for Sjögren’s Syndrome. Arthritis Rheumatol..

[226] Wijesinghe P., Xi J., Cui J., Campbell M., Pham W., Matsubara J.A. (2023). MicroRNAs in tear fluids predict underlying molecular changes associated with Alzheimer’s disease. Life Sci. Alliance.

[227] Cardoso A.G., Viltres H., Ortega G.A., Phung V., Grewal R., Mozaffari H., Ahmed S.R., Rajabzadeh A.R., Srinivasan S. (2023). Electrochemical sensing of analytes in saliva: Challenges, progress, and perspectives. TrAC Trends Anal. Chem..

[228] Nijakowski K., Owecki W., Jankowski J., Surdacka A. (2024). Salivary Biomarkers for Alzheimer’s Disease: A Systematic Review with Meta-Analysis. Int. J. Mol. Sci..

[229] Deng Q., Wong H.M., Peng S. (2024). Salivary and gingival crevicular fluid biomarkers of periodontal health and/or obesity among children and adolescents: A systematic review and meta-analysis. Heliyon.

[230] García-Carmona L., Martín A., Sempionatto J.R., Moreto J.R., González M.C., Wang J., Escarpa A. (2019). Pacifier Biosensor: Toward Noninvasive Saliva Biomarker Monitoring. Anal. Chem..

[231] Arakawa T., Kuroki Y., Nitta H., Chouhan P., Toma K., Sawada S.-I., Takeuchi S., Sekita T., Akiyoshi K., Minakuchi S. (2016). Mouthguard biosensor with telemetry system for monitoring of saliva glucose: A novel cavitas sensor. Biosens. Bioelectron..

[232] Jordaens S., Zwaenepoel K., Tjalma W., Deben C., Beyers K., Vankerckhoven V., Pauwels P., Vorsters A. (2023). Urine biomarkers in cancer detection: A systematic review of preanalytical parameters and applied methods. Int. J. Cancer.

[233] Koukourikis P., Papaioannou M., Papanikolaou D., Apostolidis A. (2023). Urine Biomarkers in the Management of Adult Neurogenic Lower Urinary Tract Dysfunction: A Systematic Review. Diagnostics.

[234] Shitanda I., Fujimura Y., Takarada T., Suzuki R., Aikawa T., Itagaki M., Tsujimura S. (2021). Self-Powered Diaper Sensor with Wireless Transmitter Powered by Paper-Based Biofuel Cell with Urine Glucose as Fuel. ACS Sens..

[235] Yuan Z.-C., Li W., Wu L., Huang D., Wu M., Hu B. (2020). Solid-Phase Microextraction Fiber in Face Mask for *in Vivo* Sampling and Direct Mass Spectrometry Analysis of Exhaled Breath Aerosol. Anal. Chem..

[236] Saifullah K.M., Rad Z.F. (2023). Sampling Dermal Interstitial Fluid Using Microneedles: A Review of Recent Developments in Sampling Methods and Microneedle-Based Biosensors. Adv. Mater. Interfaces.

[237] Abbasiasl T., Mirlou F., Mirzajani H., Bathaei M.J., Istif E., Shomalizadeh N., Cebecioğlu R.E., Özkahraman E.E., Yener U.C., Beker L. (2023). A Wearable Touch-Activated Device Integrated with Hollow Microneedles for Continuous Sampling and Sensing of Dermal Interstitial Fluid. Adv. Mater..

[238] Baker L.B. (2017). Sweating Rate and Sweat Sodium Concentration in Athletes: A Review of Methodology and Intra/Interindividual Variability. Sports Med..

[239] I Medbø J., Sejersted O.M. (1990). Plasma potassium changes with high intensity exercise. J. Physiol..

[240] Hekmat F., Kachouei M.A., Foshtomi S.T., Shahrokhian S., Zhu Z. (2023). Direct decoration of commercial cotton fabrics by binary nickel-cobalt metal-organic frameworks for flexible glucose sensing in next-generation wearable sensors. Talanta.

[241] Mo L., Ma X., Fan L., Xin J.H., Yu H. (2023). Weavable, large-scaled, rapid response, long-term stable electrochemical fabric sensor integrated into clothing for monitoring potassium ions in sweat. Chem. Eng. J..

[242] Zhang Y., Liao J., Li Z., Hu M., Bian C., Lin S. (2023). All fabric and flexible wearable sensors for simultaneous sweat metabolite detection and high-efficiency collection. Talanta.

[243] Kim J., Campbell A.S., de Ávila B.E.-F., Wang J. (2019). Wearable biosensors for healthcare monitoring. Nat. Biotechnol..

[244] Cui Y., Zhang H., Zhu J., Liao Z., Wang S., Liu W. (2022). Correlations of Salivary and Blood Glucose Levels among Six Saliva Collection Methods. Int. J. Environ. Res. Public Health.

[245] Ilea A., Andrei V., Feurdean C.N., Băbțan A.-M., Petrescu N.B., Câmpian R.S., Boșca A.B., Ciui B., Tertiș M., Săndulescu R. (2019). Saliva, a Magic Biofluid Available for Multilevel Assessment and a Mirror of General Health—A Systematic Review. Biosensors.

[246] Nguyen P.Q., Soenksen L.R., Donghia N.M., Angenent-Mari N.M., de Puig H., Huang A., Lee R., Slomovic S., Galbersanini T., Lansberry G. (2021). Wearable materials with embedded synthetic biology sensors for biomolecule detection. Nat. Biotechnol..

[247] Wang B., Yang D., Chang Z., Zhang R., Dai J., Fang Y. (2022). Wearable bioelectronic masks for wireless detection of respiratory infectious diseases by gaseous media. Matter.

[248] Srinivasan P., Dhingra K., Kailasam K. (2023). A critical insight into porous organic polymers (POPs) and its perspectives for next-generation chemiresistive exhaled breath sensing: A state-of-the-art review. J. Mater. Chem. A.

[249] Chung J., Akter S., Han S., Shin Y., Choi T.G., Kang I., Kim S.S. (2022). Diagnosis by Volatile Organic Compounds in Exhaled Breath from Patients with Gastric and Colorectal Cancers. Int. J. Mol. Sci..

[250] Li J., Hannon A., Yu G., Idziak L.A., Sahasrabhojanee A., Govindarajan P., Maldonado Y.A., Ngo K., Abdou J.P., Mai N. (2023). Electronic Nose Development and Preliminary Human Breath Testing for Rapid, Non-Invasive COVID-19 Detection. ACS Sens..

[251] de Vries R., Farzan N., Fabius T., De Jongh F.H.C., Jak P.M.C., Haarman E.G., Snoey E., Veen J.C.C.M.I., Dagelet Y.W.F., Der Zee A.-H.M.-V. (2023). Prospective Detection of Early Lung Cancer in Patients With COPD in Regular Care by Electronic Nose Analysis of Exhaled Breath. Chest.

[252] Mohan A., Pattnaik B.R., Vadala R., Bhatraju N., Rai D., Mittal S., Tiwari P., Madan K., Hadda V., Tak J. (2023). Discriminating Chronic Respiratory Diseases from Lung Cancer Using Exhaled Breath Signatures by E-Nose: A Pilot Study. Chest.

[253] Kort S., Brusse-Keizer M., Schouwink H., Citgez E., de Jongh F.H., van Putten J.W.G., Borne B.v.D., Kastelijn E.A., Stolz D., Schuurbiers M. (2023). Diagnosing Non-Small Cell Lung Cancer by Exhaled Breath Profiling Using an Electronic Nose. Chest.

[254] Chen L., Hu K., Lu M., Chen Z., Chen X., Zhou T., Liu X., Yin W., Casiraghi C., Song X. (2024). Wearable Sensors for Breath Monitoring Based on Water-Based Hexagonal Boron Nitride Inks Made with Supramolecular Functionalization. Adv. Mater..

[255] Luib E., Demleitner A.F., Cordts I., Westenberg E., Rau P., Pürner D., Haller B., Lingor P. (2023). Reduced tear fluid production in neurological diseases: A cohort study in 708 patients. J. Neurol..

[256] Shean R., Yu N., Guntipally S., Nguyen V., He X., Duan S., Gokoffski K., Zhu Y., Xu B. (2024). Advances and Challenges in Wearable Glaucoma Diagnostics and Therapeutics. Bioengineering.

[257] Shi Y., Wang L., Hu Y., Zhang Y., Le W., Liu G., Tomaschek M., Jiang N., Yetisen A.K. (2024). Contact lens sensor for ocular inflammation monitoring. Biosens. Bioelectron..

[258] Yao H., Shum A.J., Cowan M., Lähdesmäki I., Parviz B.A. (2011). A contact lens with embedded sensor for monitoring tear glucose level. Biosens. Bioelectron..

[259] Li Z., Yun J., Li X., Kim M., Li J., Lee D., Wu A., Lee S.W. (2023). Power-Free Contact Lens for Glucose Sensing. Adv. Funct. Mater..

[260] Yong C., Tracy C.R., Antes L.M., Sharp V.J.A., Antes L.M., Sanders M.L., Lockwood G.M. (2020). Urine Microscopy—Urine Made Crystal Clear. Urine Tests: A Case-Based Guide to Clinical Evaluation and Application.

[261] Pape P.T., Sharp V.J.A., Rockafellow J., Sharp V.J.A., Antes L.M., Sanders M.L., Lockwood G.M. (2020). Urine Dipstick: An Approach to Glucosuria, Ketonuria, pH, Specific Gravity, Bilirubin and Urobilinogen—Undeniable Chemistry. Urine Tests: A Case-Based Guide to Clinical Evaluation and Application.

[262] Appenheimer A.B., Ford B., Sharp V.J.A., Antes L.M., Sanders M.L., Lockwood G.M. (2020). Urine Dipstick: Urinary Nitrites and Leukocyte Esterase—Dipping into Murky Waters. Urine Tests: A Case-Based Guide to Clinical Evaluation and Application.

[263] Mann L., Antes L.M., Sanders M.L., Sharp V.J.A., Antes L.M., Sanders M.L., Lockwood G.M. (2020). Urine Dipstick: Proteinuria—Causes, Consequences and Diagnostic Approach. Urine Tests: A Case-Based Guide to Clinical Evaluation and Application.

[264] Lockwood G.M., Sharp V.J.A., Sharp V.J.A., Antes L.M., Sanders M.L., Lockwood G.M. (2020). Going with the Flow: Proper Urine Testing Methods for Clinical Practice. Urine Tests: A Case-Based Guide to Clinical Evaluation and Application.

[265] Schubbe M., Dahmoush L., Nepple K.G., Sharp V.J.A., Antes L.M., Sanders M.L., Lockwood G.M. (2020). Urine Based Tests in the Diagnosis of Genitourinary Cancers. Urine Tests: A Case-Based Guide to Clinical Evaluation and Application.

[266] Steinman J., Kuehn C., Antes L.M., Sharp V.J.A., Antes L.M., Sanders M.L., Lockwood G.M. (2020). Kidney Excretions: The Lyter Side of Urine. Urine Tests: A Case-Based Guide to Clinical Evaluation and Application.

[267] Sharp A.J., Sharp V.J.A., Sharp V.J.A., Antes L.M., Sanders M.L., Lockwood G.M. (2020). Urine Dipstick: Blood—The Spectrum of Red. Urine Tests: A Case-Based Guide to Clinical Evaluation and Application.

[268] van Abswoude D.H., Pellikaan K., Nguyen N., Rosenberg A.G.W., Davidse K., Hoekstra F.M.E., Rood I.M., Poitou C., Grugni G., Høybye C. (2023). Kidney disease in adults with Prader-Willi syndrome: International cohort study and systematic literature review. Front. Endocrinol..

[269] Yambem L., Yapici M.K., Zou J. (2008). A New Wireless Sensor System for Smart Diapers. IEEE Sens. J..

[270] Tanaka A., Utsunomiya F., Douseki T. (2015). Wearable Self-Powered Diaper-Shaped Urinary-Incontinence Sensor Suppressing Response-Time Variation With 0.3 V Start-Up Converter. IEEE Sens. J..

[271] Tajin M.A.S., Mongan W.M., Dandekar K.R. (2021). Passive RFID-Based Diaper Moisture Sensor. IEEE Sens. J..

[272] Seo W., Yu W., Tan T., Ziaie B., Jung B. (2017). Diaper-Embedded Urinary Tract Infection Monitoring Sensor Module Powered by Urine-Activated Batteries. IEEE Trans. Biomed. Circuits Syst..

[273] Li X., Zhan C., Huang Q., He M., Yang C., Yang C., Huang X., Chen M., Xie X., Chen H.-J. (2022). Smart Diaper Based on Integrated Multiplex Carbon Nanotube-Coated Electrode Array Sensors for *In Situ* Urine Monitoring. ACS Appl. Nano Mater..

[274] Wang Q., Han N., Shen Z., Li X., Chen Z., Cao Y., Si W., Wang F., Ni B.-J., Thakur V.K. (2023). MXene-based electrochemical (bio) sensors for sustainable applications: Roadmap for future advanced materials. Nano Mater. Sci..

[275] Liu X.-L., Guo J.-W., Wang Y.-W., Wang A.-Z., Yu X., Ding L.-H. (2023). A flexible electrochemical sensor for paracetamol based on porous honeycomb-like NiCo-MOF nanosheets. Rare Met..

[276] Chen K.-Y., Xu Y.-T., Zhao Y., Li J.-K., Wang X.-P., Qu L.-T. (2023). Recent progress in graphene-based wearable piezoresistive sensors: From 1D to 3D device geometries. Nano Mater. Sci..

[277] Li B., Wang C., Qin Z., Luan C., Zhan C., Li L., Lv R., Shen W., Huang Z.-H. (2023). ZnS/CuS nanoparticles encapsulated in multichannel carbon fibers as high-performance anode materials for flexible Li-ion capacitors. Energy Mater. Devices.

[278] Zhang H., Zhang D.-Z., Wang D.-Y., Xu Z.-Y., Yang Y., Zhang B. (2022). Flexible single-electrode triboelectric nanogenerator with MWCNT/PDMS composite film for environmental energy harvesting and human motion monitoring. Rare Met..

